# Inhibition of ceramide synthesis improves the outcome of ischemia/reperfusion injury in cardiomyocytes derived from human induced pluripotent stem cell

**DOI:** 10.1186/s13287-025-04340-3

**Published:** 2025-04-18

**Authors:** Pellumb Haxhikadrija, Jasmine M.F. Wu, Sascha Hübner, Katja Grün, Tom Kretzschmar, Tina Müller, Markus H. Gräler, Claudia Backsch, Anja Weise, Elisabeth Klein, P. Christian Schulze, Mohamed M. Bekhite

**Affiliations:** 1 Department of Internal Medicine I, Division of Cardiology, University Hospital Jena, Friedrich-Schiller-University, Am Klinikum 1, 07747 Jena, Germany; 2 Department of Anesthesiology and Intensive Care Medicine, Center for Molecular Biomedicine (CMB), University Hospital Jena, Friedrich-Schiller-University, Jena, Germany; 3 Department of Gynecology and Reproductive Medicine, Jena University Hospital Jena, Friedrich-Schiller-University, Jena, Germany; 4 Institute of Human Genetics, Jena University Hospital, Friedrich Schiller University, Jena, Germany

## Abstract

**Background:**

Ceramides are bioactive sphingolipids that have physiological effects on inflammation, apoptosis, and mitochondrial dysfunction. They may play a critical role in the harm of ischemia/reperfusion (IR). Ceramides and IR injury are not well-studied, and there is a lack of human data.

**Methods and results:**

Current studies aimed to investigate the role of ceramide buildup in cardiomyocytes (CMs) death using CMs derived from human induced pluripotent stem cell (hiPSC) as a model for simulating IR injury in vitro. In our model, serum- and glucose-free media was used to expose hiPSC-derived CMs to hypoxia (3% O_2_) for 6 h (hrs), followed by reoxygenation (20% O_2_) for 16 h. In contrast to normoxia (control) or hypoxia (ischemia), our data showed that following IR, there was an increase in the formation of mitochondrial superoxide and the mRNA levels of genes regulating ceramide synthesis, such as *CerS2* and *CerS4* in CMs. Further, there was a considerable rise in the levels of total ceramide, long-chain (C16:0, C18:0, and C18:1), and very long-chain (C22:0 and C24:1) ceramide species in CMs following reperfusion in comparison to control or ischemic CMs. Interestingly, compared to CMs exposed to IR without inhibitor, our data showed that inhibition of ceramide formation with fumonisin B1 (FB1) significantly lowered ceramide levels, reduced apoptosis, improved mitochondrial function, and enhanced survival of CMs exposed to IR. Furthermore, we used a transgenic mouse model, in which the CerS2 gene was overexpressed in the CMs of α-MHC-CerS2 mice, to validate the basic idea that ceramide contributes to heart disease in vivo. Our results showed that the heart tissues of α-MHC-CerS2 mice had significant levels of long-chain and very long-chain ceramides, which causes increased apoptosis, proinflammatory cytokines, interstitial inflammatory cell infiltration, and collagen deposition.

**Conclusions:**

Results from both in vitro and in vivo experiments show that ceramides have a significant role in either mediating or inducing damage to CMs. Additionally, in vitro findings show that ceramide reduction improves the outcome of IR injury by lowering intracellular Ca^2+^ [Ca^2+^]_i_ concentration and improves mitochondrial function changes during IR.

**Supplementary Information:**

The online version contains supplementary material available at 10.1186/s13287-025-04340-3.

## Introduction

After tissue ischemia e.g., a myocardial infarction, the main treatment objective is to restore circulation in the affected ischemic area, a process called reperfusion. However, even though it is the only way to treat ischemia, reperfusion leads to further significant damage in the tissues, a phenomenon called reperfusion injury, which was first described by Jennings in an IR injury model in dogs [1]. Reperfusion injury occurs also after iatrogenic-induced cardioplegia during major heart operations [2]. A major problem in every reperfusion therapy is the so-called reperfusion injury, which often leads to additional myocardial damage [3]. However, the lack of consistency between the basic research and clinical trials leads to more information being needed to better understand the reperfusion itself, particularly in humans, and its possible alleviation [3].

Myocardial biopsy has a lot of risks because of its invasiveness, and primary human culture remains a difficult task [4]. In 2006, Yamanaka and his colleagues discovered the reprogrammed somatic cells into induced pluripotent stem cells (iPSC) using four genes: OCT4, SOX2, KLF4, and c-MYC (OSKM; also known as Yamanaka factors) from adult mouse fibroblast [5], and in 2007 from human fibroblast [6]. Interestingly, Raghavan in 2024 showed that the small, nontoxic molecules Metadichol can stimulate the production of Yamanaka factors in triple-negative primary breast tumors (HCAF-TNPBC) cells, fibroblasts, and A549 as well as Colo-205 cancer cells [7]. By using Metadichol, we can avoid the need for viruses to deliver the transcription factors, which are linked to tumorigenesis [8], provide a way for direct testing on humans, and potentially use in stem cell therapy [7]. This technology enables us to differentiate any body cell line from hiPSC to investigate physiology and diseases in vitro [9–14]. It has been shown, that cardiomyocytes (CMs) derived from hiPSC display normal structure and physiological properties as well as, react to cholinergic/adrenergic stimulation [15–17]. Therefore, hiPSC-derived CMs could be used for modeling specific genetic diseases [18], and non-genetic conditions like cardiac physiology in diabetic cardiomyopathy [19] or IR injury [20].

Multiple factors play a role in IR injury, e.g., reactive oxygen species (ROS) and intracellular Ca^2+^ [Ca^2+^]_i_ accumulation. ROS is one of the main factors involved in IR injury [21, 22]. It has been shown, that mitigation of ROS with e.g., propofol [23], mitoquinone mesylate (MitoQ) [24], overexpressed superoxide dismutase (SOD) [25] or pyruvate [26] improves IR outcome on a cellular level. Also, there are data linking Ca^2+^ accumulation and reperfusion injury. Accumulation of Ca^2+^ in mitochondria after initiation of reperfusion was observed [27]. During reperfusion there is an increased frequency and amplitude in Ca^2+^ oscillations, leading to a hypercontracture state. Its attenuation with e.g. inhibition of sarcoplasmic/endoplasmic reticulum Ca^2+^ ATPase 2a (SERCA2a) or ryanodine receptor (RyR) led to a reduction in this hypercontracture state and lesser injury [28, 29].

Ceramides are the combination of a sphingosine backbone with an attached fatty acid (FA) of variable length. They are known for their biological effects on many signaling pathways like apoptosis, inflammation, and mitochondrial dysfunction [30, 31] Pathways. Three synthesis pathways are known: the *de novo* pathway, the hydrolysis of sphingomyelin, and the salvage pathway. Ceramides also play a role in IR. Increased levels of ceramides were detected in the apoptotic myocardium of rat hearts exposed to IR [32]. After a transient occlusion of the left anterior descendent artery of the heart of mice, one of the key enzymes of *de novo* pathway serine-palmitoyltransferase long chain base subunit 1 (SPTLC1) was higher in the area at risk. This was attenuated in samples treated with *de novo* synthesis pathway inhibitor myriocin [33]. Also, fumonisin B1 (FB1), a non-specific ceramide synthase (CerS) inhibitor involved in *de novo* and salvage pathway of ceramide synthesis was studied in an IR model of the brain [34]. Evidence from clinical studies also suggests that elevated ceramide levels are predictors of coronary artery disease [35] and its outcome [36, 37]. These findings suggest a detrimental link between increased ceramides and cardiovascular disease. The mitigation of ceramides could be beneficial for the treatment of different heart failure phenotypes, including IR injury. The goal of our study was to investigate the underlying effects of increased ceramides levels on hiPSC-derived CMs exposed to IR, and if ceramide reduction improves IR outcome.

## Methods

### Reprogramming of fibroblasts to human induced pluripotent stem cell (hiPSC)

After human foreskin fibroblast cell (HFF-1-SCRC-1041, from ATCC) were reprogrammed into hiPSC-FS.2, the cell line was registered to the human pluripotent stem cell registry (hPSCreg) and received a unique reference code (UKJi005-A). The UKJi005-A cell line was generated by using non-integrating Sendai virus (SeV) vectors (CytoTuneTM-iPS 2.0 Sendai Reprogramming Kit, Life Technologies A1378001) at a multiplicity of infection (MOI) of KOS: c-MYC: KLF4 (5: 5: 3). Following three weeks of transduction, the proper hiPSC colony size was selected and expanded for ten passages to confirm the absence of the reprogramming vectors by RT-PCR as shown in supplementary material Fig. S1A.

### Maintaining of HiPSC

The UKJi005-A cell line was cultured, without feeder cells, on 1:80 diluted matrigel (BD Biosciences, Heidelberg, Germany) coated flask 75 cm^2^ (BD Biosciences, Heidelberg, Germany) in essential 8 (E8) (Thermo Fisher Scientific, Darmstadt, Germany). The medium was changed every day. They were split using a ReLeSR dissociation reagent (STEMCELL technologies, Cologne, Germany) once they reached ∼ 80% confluency. The cells were dissociated using 3 ml of ReLeSR dissociation reagent for 7 minutes (min) at 37 °C after first being washed with phosphate-buffered saline (PBS) free of calcium and magnesium. Following the collection of the cells using 5 ml pipettes, 3 ml of E8 media was added, and the cells were centrifuged at 900 rpm for 5 min. The cells were suspended in 12 milliliters of E8 media and plated on matrigel-coated 12-well plates for further differentiation. During splitting of the cells, 10 μM of the ROCK inhibitor Y-27,632 (STEMCELL technologies) was added to the E8 medium.

### Embryoid bodies (EBs) formation

EBs were generated by aggregating the cells, using ultra-low attachment plates, in an E8 medium containing 10 μm ROCK inhibitor Y-27,632 (STEMCELL technologies) for two days on an orbital shaker. Cell aggregates were then further cultured in DMEM medium (Thermo Fisher Scientific) containing 20% fetal bovine serum (FBS) (Merck, Darmstadt, Germany), 2 mM L-glutamine, 55 μM ß-mercaptoethanol, and 1x non-essential amino acids (Merck). The differentiation medium was changed every other day for 3 months. Spontaneous differentiated EBs were fixed in formalin and processed according to standard procedures for paraffin embedding and hematoxylin/eosin staining (Roth, Lichtentanne, Germany).

### Differentiation of HiPSC to cardiomyocytes (CMs)

We have established a protocol to produce high-yield CMs (∼ 80%) in 8 days from multiple hiPSC lines without cell sorting or selection [19, 38]. In brief, at day 0 RPMI medium (Thermo Fisher Scientific) was supplemented with 4 ng/ml bone morphogenetic protein 4 (BMP4) (R&D Systems, Wiesbaden, Germany), 5 ng/ml activin-A (eBioscience, San Diego, CA), 2 μM CHIR99021 (Axon Medchem BV, Groningen, Netherlands) and 1% B-27™ was added to fully confluent hiPSC. For the next two days, the RPMI medium was supplemented with 4 ng/ml BMP4 (R&D Systems), 2% B-27™ (Thermo Fisher Scientific), and 2 μM IWP-2 (Santa Cruz Biotechnologies, Santa Cruz, CA). Three days later, the cells were then cultured in RPMI medium (Life Technologies) supplemented with 256 μg/ml ascorbic acid (Merck) and 2% B27™ (Thermo Fisher Scientific). Starting from day 14, the cells were incubated in KnockOut DMEM medium (Thermo Fisher Scientific) enriched with 3% KnockOut™ Serum Replacement (Thermo Fisher Scientific), L-Glutamine 200 mM (Merck), and Penicillin-Streptomycin (10000 U/ml and 10 mg/ml, Merck). The cells metabolically matured around day 28 when we began our experiments [38].

### Mimicking ischemia/reperfusion model in vitro using hiPSC-derived CMs

To mimic ischemia/reperfusion (IR) injury we modified the model developed by Wei et al. [39]. 4-weeks-old CMs were washed with PBS and incubated in serum- and glucose-free DMEM medium (Thermo Fisher Scientific) in 3% O_2_ and 5% CO_2_ in an incubator for 6 h, then the cells were incubated in an incubator with normal conditions (20% O_2_, 5% CO_2_) and with glucose-rich maintenance DMEM medium enriched with 3% serum replacement (Thermo Fisher Scientific), L-Glutamine (Merck) and Penicillin-Streptomycin (Merck). During reperfusion, experiments (e.g. ROS and Ca^2+^ measurements) were made after 2–4 h of reperfusion, and the experiments (viability, ceramide level measurements, mRNA level analysis) were made after 16 h. Starting from ischemia, each medium that was changed contained the given concentration of the fumonisin B1 (FB1) inhibitor (VWR Chemicals).

### Animals

We used a transgenic mouse model, based on the tetracycline-controlled Tet-on system [40], where the CerS2 gene was overexpressed in the CMs of α-MHC-CerS2 mice. The mice were generated and kindly provided by the Columbia University Medical Center (630 West 168th St. New York, NY 10032, 212-305-CUMC) and implemented in our lab using embryo transfer technology after the study protocol was approved on July 13, 2020, under the title “Influence of elevated ceramide levels on heart damage and systemic metabolism” by the appropriate State Office of Food Safety and Consumer Protection (TLLV, Bad Langensalza, Germany, Local registration number: UKJ-20-010). The work has been reported in line with the ARRIVE guidelines 2.0. All experiments were conducted according to the National Institute of Health Guidelines for the Care and Use of Laboratory Animals (8th edition), to the European Community Council Directive for the Care and Use of Laboratory Animals of 22 September 2010 (2010/63/EU), the current version of the German Law on the Protection of Animals and the guidelines for animal care. The MHC-Tet3G mice were mated with pTRE3G-CerS2 mice to generate α-MHC-CerS2 mice (supplementary material methods). CerS2 is only expressed in CMs of MHC-CerS2 mice after a month of doxycycline (0.545 mg/g) supplementation. (supplementary material Fig. S2B, C). The administration of doxycycline can regulate the specific overexpression of CerS2 locally and temporarily in the heart. C57BL/6J wild-type mice were used as a control group. All animals had ad libitum access to food and water and were exposed to controlled light/dark cycles during the experiment. Further, all mice were weighed and examined every two days to monitor their clinical status via scoring system proposed recently (UKJ-20-010). The scoring system included the observation of body weight, physical and social behavior, and discomfort did not show the difference between control and MHC-CerS2 mice. Both genders of mice were employed in this investigation, and all were 8 weeks old at the beginning of the experiment. After a month of doxycycline feeding, mice were euthanized by cervical dislocation, and the hearts were extracted. The materials for RNA, protein, mass spectrometry, immunofluorescence, and histology were obtained by dividing the weighted hearts into separate pieces. After being shock-frozen in liquid nitrogen, the samples were then stored at -80° C.

### Immunofluorescence staining

Differentiated EBs were plated on cover glass coated in matrigel for 14 days, and then the EBs were fixed using methanol and acetone (7:3) for 30 min at -20 °C. Next, 0.1% Triton X-100 (PBST) was used to permeabilize the cells for 10 min. For immunofluorescence, 4 μm-thick tissue sections from cryostat or paraffin-embedded tissue samples were used. The corresponding primary antibody (supplementary material table S1) was diluted in 10% milk with 0.01% PBST. After using the primary antibodies, the secondary antibodies (supplementary material table S1) were added for 1 h at room temperature. Following the removal of the secondary antibody, nuclei were stained with 5 μg/mL Hoechst 33342 (Invitrogen/Thermo Fisher Scientific). Immunofluorescence was examined using a confocal laser scanning microscope 900 (cLSM 900; Zeiss, Jena, Germany). To perform the immunofluorescence analysis, Zen Blue 3.0 software was used (Zeiss).

### Alkaline phosphatase staining

The pluripotency of hiPSC colonies was detected using an alkaline phosphatase staining (ALP) kit (Abcam) according to the manufacturer’s protocol.

### Karyotype analysis

UKJi005-A cell was treated with 10 μg/ml Colcemide for 2.5 h to arrest cells in metaphase. UKJi005-A cell line was trypsinized with ReLeSR (STEMCELL technologies) and transferred into the falcon with medium for centrifugation. The cells were treated with 0.075 M KCl for 10 min before preserved in freshly prepared methanol: acetic acid (3:1). The fixed metaphase chromosomes were stained with Giemsa after trypsin treatment to produce a repeatable black and white banding pattern. Subsequently, the metaphases were imaged by Metafer4 (Version 3.14.4 and CoolCube1 camera; Metasystems, Altlußheim, Germany) and analyzed with the Ikaros software (Version 5.9.2 CM, Metasystems, Altlußheim, Germany).

### Real‑time‑polymerase chain reaction (qPCR)

Total RNA from CMs were isolated and prepared using QIAshredder columns and an RNeasy mini kit (Both from QIAGEN, Hilden, Germany) according to instructions in the user manual. Following the RNA isolation process, we created cDNA using the RevertAid First Strand cDNA Synthesis Kit (Thermo Fisher Scientific). Lastly, qPCR amplification was performed using the primer sets as shown in supplementary material table S2. Relative expression values were calculated by normalizing the CT values of the target gene to that of the internal standard gene *GAPDH* as the normalizer, using the ΔΔCT-method.

### Western blot analysis

CMs were lysed using the previously described protocol [41] in an ice-cold lysis buffer. 8%, 12%, and 4–20% gels were used to separate protein aliquots (20 μg protein/lane) before they were transferred onto PVDE membranes and detected with rabbit- Anti-SERCA2 ATPase, Anti-Ryanodine receptor 2 (RYR-2), Anti-Bcl-2, and Anti-Bax followed by incubation with HRP (supplementary material table S1). The images were taken with a LAS 3000 digital imaging technology system (Fujifilm, Tokyo, Japan).

### Measurement of intracellular Ca^2+^ [Ca^2+^]_i_ transient levels

One week before the measurement upon their maturation (day 21) the cells were split using 2% collagenase type IV (SERVA, Heidelberg, Germany) and were plated in 24-well glass bottom plates (Sarstedt, Nümbrecht, Germany). 4-weeks-old CMs were exposed to ischemia or IR or just changing the maintenance mediums in normoxia (control) samples, cells were washed with PBS and incubated with 5 μM fluo-4-AM (Molecular Probes, Karlsruhe, Germany) diluted in serum-free maintenance medium for 15 min and was examined using a cLSM 900 (Zeiss). From each experiment, 3–5 areas of interest in a CMs conglomerate were chosen. Data were analyzed with Zen Blue 3.0 software (Zeiss), and the results are depicted as an average of at least three independent experiments.

### Measurement of mitochondrial superoxide levels

Single cells of CMs were plated in 24-well glass bottom plates. After inducing ischemia or IR, cells were washed with PBS and incubated with 5 μM MitoSOX (Thermo Fisher Scientific), the mitochondrial superoxide indicator, diluted in serum-free maintenance medium for 15 min and examined using cLSM 900 (Zeiss). From each experiment, 3–5 areas of interest in a CMs conglomerate were chosen. Data were analyzed with Zen Blue 3.0 software (Zeiss).

### Flow cytometry analysis

CMs were dissociated with TrypLE (GIBCO, Karlsruhe, Germany) and filtered through a 40-μm cell strainer. Cells were then fixed with 4% paraformaldehyde (PFA), pH 7.4, dissolved in PBS, and permeabilized using 0.1% PBST. The cells were then incubated with primary antibody (supplementary material table S1) for 30 min. The samples were washed with 2% FBS in PBS, followed by incubation with secondary antibody (supplementary material table S1) at 1:500 for 20 min. The cells were rinsed with 2% FBS. The analyses were carried out using a FACS Calibur (BD, Heidelberg, Germany).

### Vitality measurements

Propidium iodide (PI) was used to stain dead cells, while fluorescein diacetate (FDA) was used to stain living cells to evaluate vitality. Single cells of CMs were plated in 24-well glass bottom plates. Following ischemia or IR induction, cells were rinsed with PBS, treated with 10 μM PI, and prepped with 5 μm FDA in DMSO before being analyzed with a cLSM 900 (Zeiss). The second test for living cells was the PrestoBlue assay (Thermo Fisher Scientific). Before being tested, single cells were plated on 96-well (3 × 10^4^ cells/well) Sarstedt microplates in a maintenance medium. The cells were then maintained at 37 °C in a humidified atmosphere with 5% CO_2_ for at least 72 h. After ischemia or IR induction, cells were treated with PrestoBlue reagent and subsequently analyzed using a Plate-Reader (EnSpire Multimedia Plate-Reader). Each experiment was repeated three times and each experiment had an average 8 replicates.

### Measurement of intracellular ceramide level

Ceramide levels were measured as previously described [42, 43]. The Prominence LC-20 A Modular HPLC System (Shimadzu, Duisburg, Germany) coupled with the QTrap triple-quadrupole mass spectrometer (Sciex, Darmstadt, Germany) equipped with an APCI source operating in positive mode was used. Standard solutions were acquired from Avanti Polar Lipids Inc. (Alabaster, AL, USA) and added for ceramide identification. The analytical results were quantified with Analyst 1.6.3 (Sciex) based on external standard curves.

### Seahorse metabolic profiling

Mitochondrial respiration and a complete bioenergetics profile were evaluated using a Seahorse *XF96* analyzer (Seahorse Biosciences/Agilent Technologies, Waldbronn, Germany). The oxidative phosphorylation was measured through the oxygen consumption rate (OCR) and glycolysis was measured through the extracellular acidification rate (ECAR). The CMs as single cells were plated on *XF96* microplates (Seahorse Biosciences/Agilent Technologies) at 3 × 10^4^ cells/well in the media. The CM respiration was assayed under basal conditions and after addition of inhibitor of carnitine palmitoyltransferase-1 (CPT-1) etomoxir (40 μM) and electron transport chain inhibitors as following: mitochondrial inhibitor oligomycin (2 μM), mitochondrial uncoupler carbonylcyanide p-trifluoromethoxyphenylhydrazone (FCCP; 0.3 μM), and a respiratory chain inhibitor antimycin A (0.5 μM) and rotenone (0.5 μM). These concentrations were selected based on the Agilent Seahorse FA-oxidation substrate assay and Mito Stress test Kit protocol (Seahorse Biosciences/Agilent Technologies). Respiratory parameters for values were quantified according to Seahorse Biosciences software.

### Statistical analysis

The results were presented as an average of at least three to four independent experiments. Data are presented as mean values ± SD. GraphPad Prism 8 (GraphPad Software Inc., San Diego, USA) was applied for unpaired and paired t-test and one-way ANOVA. The *p*-value < 0.05 was considered significant. The level of significance is illustrated by * at *p* < 0.05, ** at *p* < 0.01, and *** at *p* < 0.001 in graphical representations.

## Results

### Characterization of UKJi005-A cell line after reprogramming HFF-1 cell

**Fig. 1:**
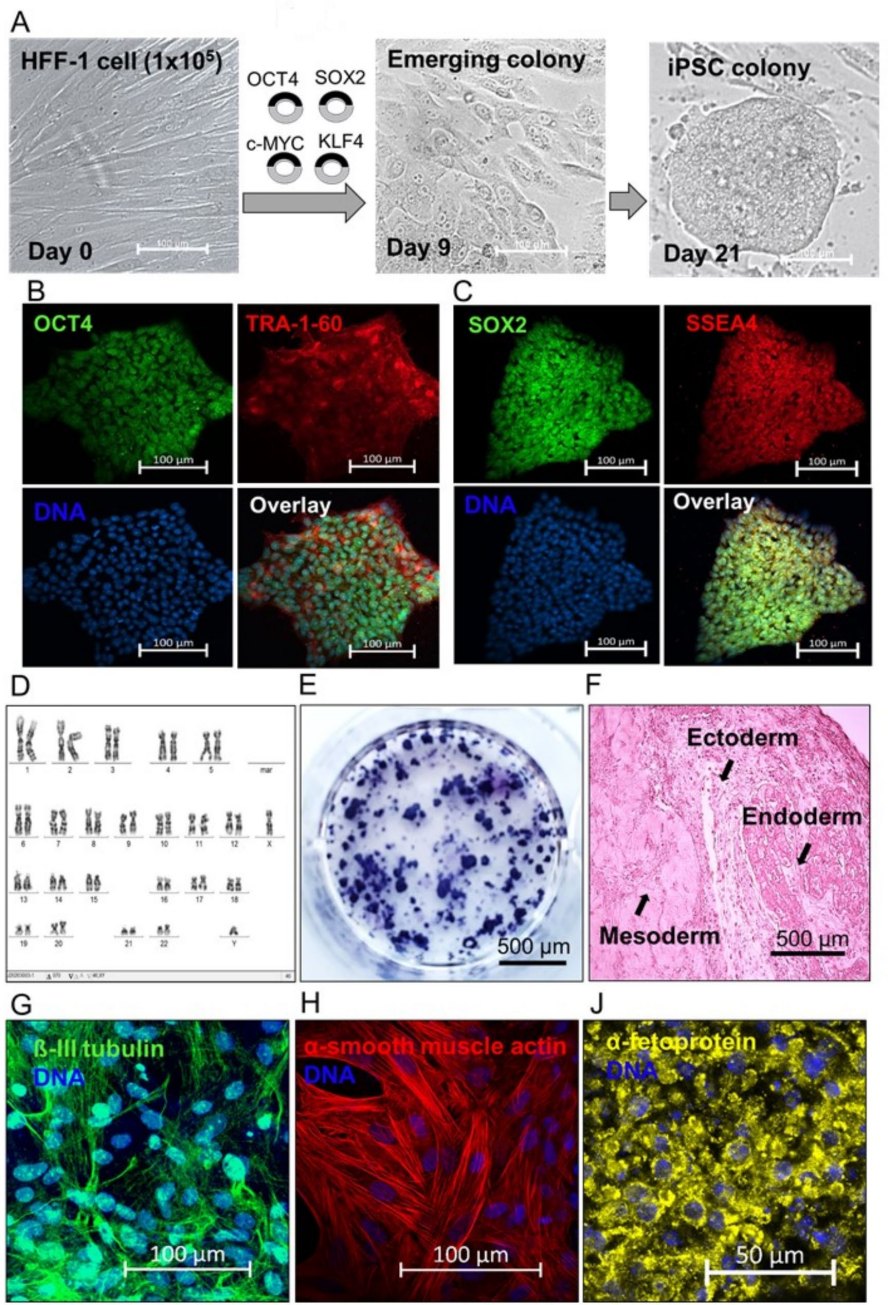
Key pluripotency markers of hiPSC-FS.2 (UKJi005-A) generated from reprogramming human foreskin fibroblast (HFF-1) cells. (**A**) Phase-contrast image of HFF-1 cell before reprogramming and hiPSC colony formation after 3 weeks of HFF-1 cell transduction; scale bar = 100 μm. (**B** and **C**) Embryonic stem cell morphology and pluripotency markers OCT4, TRA1-60, SOX2, and SSEA4 in 4-weeks-old iPSC colony. Nuclei were stained with Hoechst 33,342 (blue), and the scale bar = 100 μm. (**D**) Karyotyping shows the genomic integrity in hiPSC after reprogramming. (**E**) Alkaline-phosphatase of UKJi005-A cell line, and (**F**) Hematoxylin and eosin staining of embryoid body section revealed differentiation of ectoderm (neuroectoderm), mesoderm (muscles), and endoderm (gut epithelium). (**G**) Neuron stained by ß-III tubulin accounted for ectoderm, (**H**) smooth muscle by α-smooth muscle actin accounted for mesoderm, and (**J**) fetal liver cells by α-fetoprotein accounted as an endodermal marker in plated embryoid bodies. Nuclei were stained with Hoechst 33,342 (blue), and the scale bar = 100 μm

HiPSC was generated from HFF-1 cell using SeV carrying the four Yamanaka factors (*OCT4*,* SOX2*,* KLF4*,* and c-MYC*) [6]. Following a successful reprogramming process and around day 21, the UKJi005-A cell line exhibits the typical shape of an embryonic stem cell colony (Fig. 1A). After reprogramming, the UKJi005-A cell line harboring the transcription factors exhibits immunofluorescence for the expression of OCT4, TRA-1-60, SOX2, and SSEA4 (Fig. 1B, C). When comparing the UKJi005-A cell line to the HFF-1 cell, RT-PCR analysis revealed increased endogenous pluripotent gene expression in *OCT4*, *Nanog*, and *SOX2* (supplementary material Fig. S1B). After reprogramming, karyotyping showed the genomic integrity in reprogrammed UKJi005-A cell line (Fig. 1D). Furthermore, the UKJi005-A cell’s pluripotency was verified by staining the colony with alkaline phosphatase (Fig. 1E) and examining how the cells differentiated into three germ layers by forming ectoderm, mesoderm, and endoderm structures in sections of embryoid bodies stained with hematoxylin and eosin (Fig. 1F). The pluripotency was further validated by staining the neuron with ß-III tubulin accounted for ectoderm; smooth muscle by α-smooth muscle actin accounted for mesoderm, and fetal liver cells by α-fetoprotein accounted as an endodermal marker in sections of embryoid bodies (Fig. 1G-J). Taken together, these results demonstrated the successful reprogramming of HFF-1 cells to pluripotent UKJi005-A cell line.

### Cardiomyocytes (CMs) differentiation from UKJi005-A cell line

**Fig. 2:**
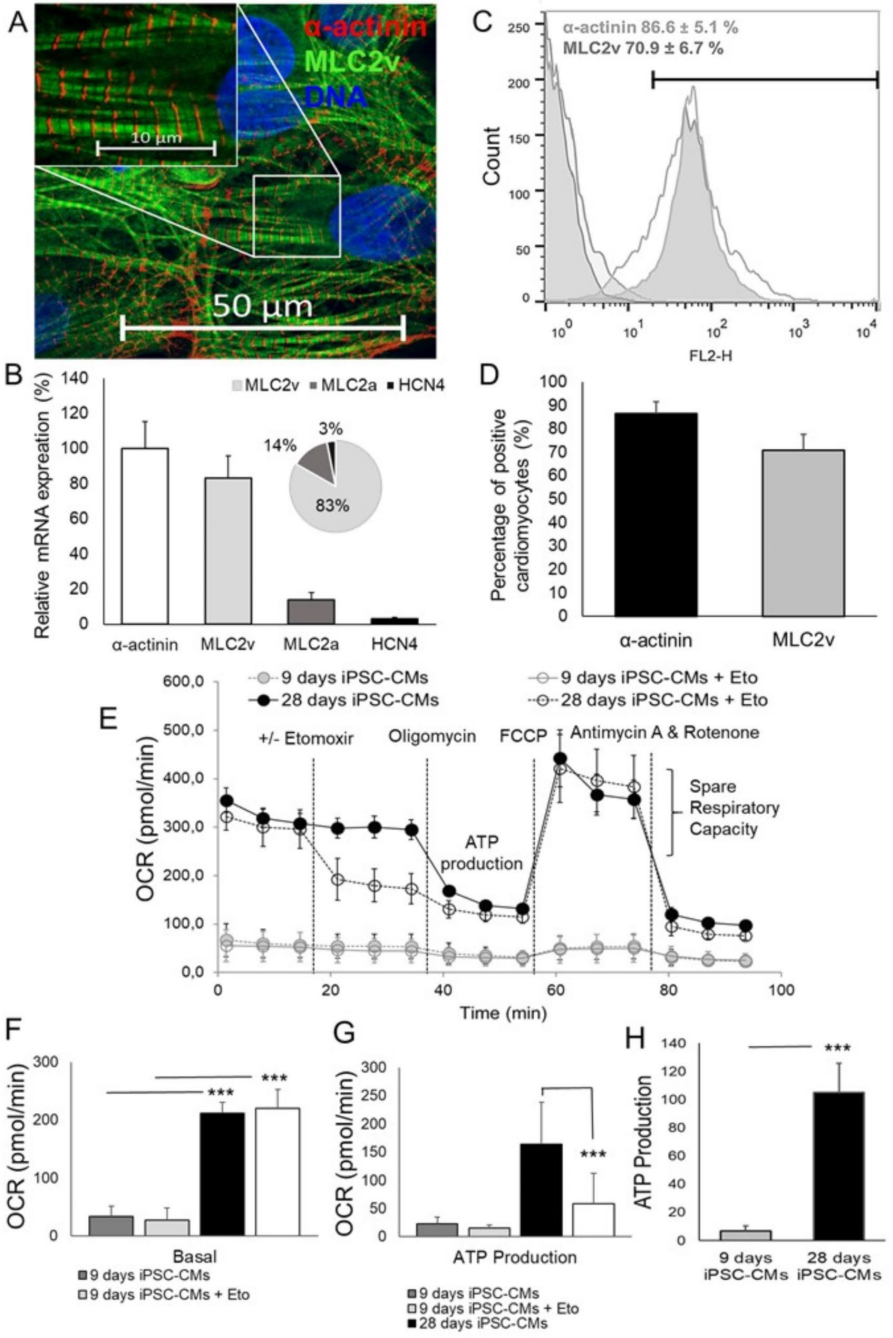
Characterization of cardiomyocytes (CMs) derived from hiPSC-FS.2 (UKJi005-A). (**A**) Immunofluorescence image of the cardiac sarcomeric muscle markers α-actinin (red) and MLC2v (green) in CMs following 28 days of differentiation. Nuclei were stained with Hoechst 33342 (blue; *n* = 3. (**B**) Subtype-specific CMs were studied by the gene expression in differentiated CMs. The mRNA expression level of *MLC2v* showed ventricular-like CMs, *MLC2a* for atrial-like CMs, *HCN4* for sinoatrial node-like cells, and pacemaker-like CMs as well as cardiac sarcomeric muscle markers *α-actinin*. (**C**) Flow cytometry analysis of cardiac marker α-actinin and MLC2v expressed at day 28. (**D**) Graphs show the percentages of positive CMs. Data are representative of *n* = 4 independent experiments and values are expressed in mean ± SD. Efficacy of mitochondrial function in 28-days-old CMs. (**E**) Assays of mitochondrial respiration and mitochondrial β-oxidation in 28-day-old CMs before and after adding pharmacological agents using a *Seahorse XF96 analyzer*; *n* = 3. (**F**) Increased mitochondrial basal respiration (**G**), significant increase in FA β-oxidation of 28-days-old CMs were determined by using the CPT1 inhibitor etomoxir (Eto) treated and non-treated CMs; *n* = 3. (**H**) ATP production in the 28-day-old CMs compared to premature 9-day CMs. Data are representative of *n* = 3 independent experiments and values are expressed in mean ± SD. *** for *p* < 0,001

Using conditions protocol, our results suggest CMs can differentiate efficiently from UKJi005-A cell line and start beating around day 7 (74.5 ± 7.9 beats/min). To confirm the success of CM differentiation, we stained the CMs with sarcomeric muscle marker α-actinin and myosin light chain 2 isoform (MLC2) for ventricle (MLC2v) CM (Fig. 2A, B). As shown in Fig. 2A, sarcomeric pattern α-actinin was well-organized into an interconnected of myofibrils in 28-day-old CMs. Gene expression analysis of CMs structural components showed that the level of MLC2v represented 83.2 ± 12.7%, which is higher than MLC2a 13.7 ± 5.1%, and expression of pacemaker hyperpolarization-activated cyclic nucleotide-gated 4 channels (HCN4) 3.1 ± 0.74% from total α-actinin level in 28-days-old CMs (Fig. 2B). Additionally, flow cytometry analysis revealed that the percentage of α-actinin positive cells represented 86.6 ± 5.1% (*n = 4*) after 4 weeks of CMs differentiation and MLC2v positive cells represented 70.9 ± 6.7% (*n = 4*) from the total number of cells (Fig. 2C, D). To verify the metabolic maturity of CMs produced from UKJi005-A cell line, ß-oxidation, mitochondrial function, and a comprehensive bioenergetic profile were analyzed (Fig. 2E). We observed a significant increase in basal respiration (Fig. 2F) and ATP production, through measurements of the difference in OCR of etomoxir-treated and non-treated CMs, indicating an increase in depends on mitochondrial ß-oxidation in the 28-days-old CMs compared to early 9-days-old CMs (105.2 ± 20.5 vs. 6.76 ± 3.82, *p* < 0.001) to fulfill the needs of mature CMs activity (Fig. 2G, H). These results confirm the ability of UKJi005-A cell line to differentiate into mature CMs and provide unique in vitro human CMs for the understanding of the physiology and metabolic function of diseases.

### IR injury leads to CMs apoptosis and leads to an additional decrease in viability

**Fig. 3:**
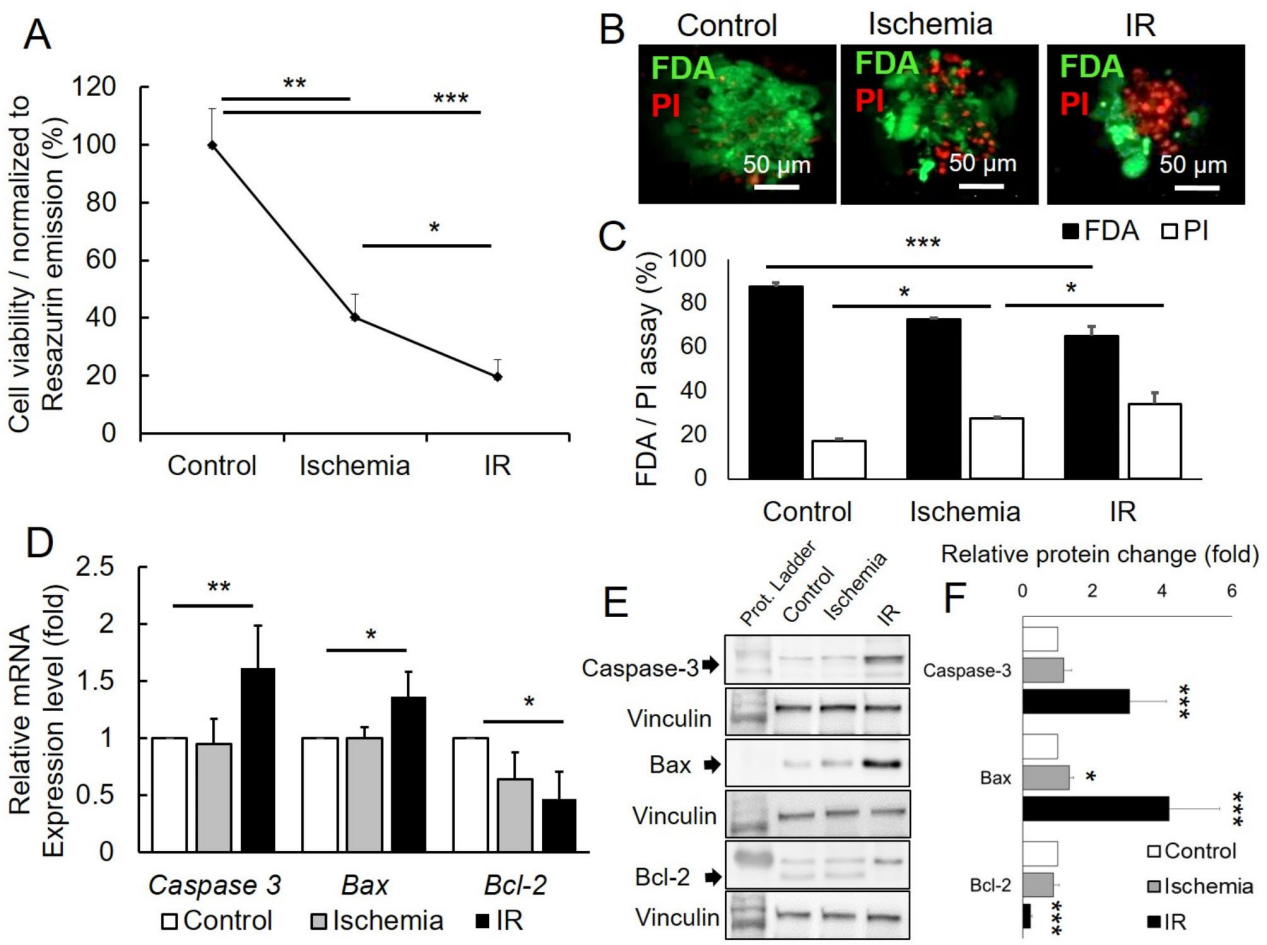
Reperfusion reduces viability in cardiomyocytes (CMs) exposed to hypoxia/ischemia (3% O_2_) in serum- and glucose-free medium for 6 h and then subsequently to reoxygenation/reperfusion (20% O_2_) and further incubation in normal conditions medium containing serum- and glucose for 16 h. (**A**) Graphic representing viability tested with PrestoBlue assay in baseline, after ischemia, and after IR; *n* = 3. (**B**) Representative immunofluorescence images of normoxia (control), ischemia (hypoxia), and IR-CMs after having stained with fluorescein diacetate (FDA) and propidium iodide (PI) live cells and dead cells respectively; scale bar = 50 μm. (**C**) Graphic representation of mean percentage of viability of CMs using PI for dead cells and percentage of FDA for live cells; *n* = 3. (**D**) Fold levels of mRNA for genes regulate cell apoptosis (*Caspase 3*,* Bax*,* and Bcl-2*) in CMs exposed to ischemia and IR compared to control CMs; *n* = 3. (**E** and **F**) Representative western blot results of comparison of Caspase-3, Bax, and Bcl-2 protein level in CMs exposed to ischemia and IR compared to control CMs; *n* = 3. The values were expressed as mean ± SD. Comparisons between groups were made using the paired t-test for PrestoBlue, FDA/PI assay, and the unpaired t-test for qPCR. * for *p* < 0.05, ** for *p* < 0.01; *** for *p* < 0,001 *n* = 3

During ischemia, certain damage to the myocardial tissue occurs, and after CMs reperfusion, a further decrease in viability in most of the cases is observed [1]. Our in vitro model of hypoxia/reoxygenation was established based on the in vitro model of Wei et al. [39] with slightly modified exposure times. The terms ischemia and reperfusion are not ideally applicable for monolayer cell cultures and are dominantly used for tissues with actual vasculature up on its disruption (ischemia) and its subsequent restoration (reperfusion). Hence from here on we will refer to conditions in hypoxia (3% O_2_) and glucose- and serum-free medium as ischemia and the subsequent restoration of normoxia (20% O_2_) and glucose- and serum-rich medium as reperfusion. First, we established the optimal exposure time towards hypoxia and subsequent reoxygenation to determine the significantly lower viability of CMs after IR in comparison to ischemia alone. Our results revealed that exposure of CMs derived from UKJi005-A to ischemia for 6 h and subsequent reperfusion for 16 h yielded the optimal results. After CMs were exposed to 6 h of ischemia, the viability of the cells decreased to 40.1 ± 8.3% (*p < 0.01*) as detected by Presto-Blue assay (Fig. 3A). However, after 16 h of CMs reperfusion, the viability of cells decreased significantly to 19.6 ± 5.9% (*p* < 0.05) (Fig. 3A). However, no significant change in the presence of DMSO in terms of viability compared to its absence (supplementary material Fig. S3). A similar impact in viability was detected with immunofluorescence staining of live cells with FDA (87.55 ± 1.74% vs. 72.5 ± 0.6%, *p* < 0.001) and dead cells with PI (17.38 ± 0.67% vs. 27.68 ± 0.4%, *p* < 0.05) for 6 h of ischemia (Fig. 3B, C). However, after 16 h of CMs reperfusion, the live cells decreased to 65.01 ± 4.05% (*p* < 0.001) (Fig. 3B, C). Furthermore, gene expression and protein level of caspase-3 and apoptotic gene Bax were detected via qPCR and western blot in CMs after 6 h of ischemia followed by 16 h of reperfusion in comparison to respective normoxia (control) at gene expression (1.61 ± 0.38 vs. 1.0, *p* < 0.01) and (1.36 ± 0.22 vs. 1.0, *p* < 0.05), as well as protein level was (3.06 ± 1.05 vs. 1.0, *p* < 0.001) and (4.19 ± 1.2 vs. 1.0, *p* < 0.001) respectively (Fig. 3D-F). Finally, a lower expression of anti-apoptotic gene BCL-2 (0.47 ± 0.24 vs. 1.0, *p* < 0.05) and protein level (0.27 ± 0.15 vs. 1.0, *p* < 0.001) in IR-CMs was also detected compared to control (Fig. 3D). These results suggest that this way underlying triggered apoptosis could be the way of reduction in viability caused by reperfusion.

### Alterations in [Ca^2+^]_i_ and mitochondrial superoxide in CMs are linked to IR injury

**Fig. 4:**
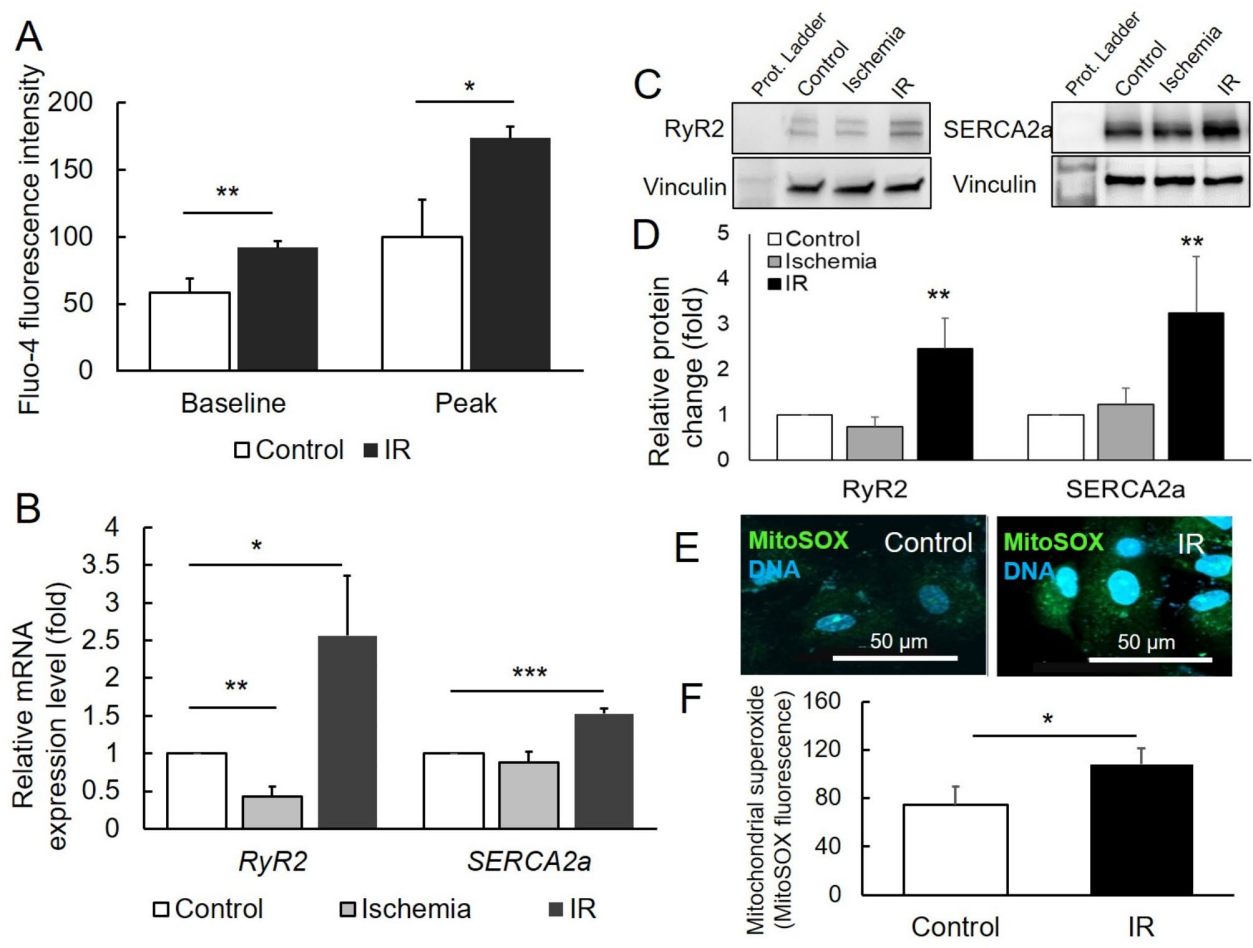
Level of intracellular Ca^2+^ [Ca^2+^]_i_ of both baseline and peak as well as mitochondrial superoxide in cardiomyocytes (CMs) during ischemia/reperfusion (IR) injury. (**A**) levels of [Ca^2+^]_i_ of CMs at baseline (in resting), as well as peak (in contracting), detected with fluo-4 dye after exposure to hypoxia/ischemia (3% O_2_) in serum- and glucose-free medium for 6 h and then subsequently to reoxygenation/reperfusion (20% O_2_) and in normal conditions medium containing serum- and glucose for 16 h. (**B**) Fold levels of genes involved in influx (*SERCA2a*) and outflux (*RyR2*) of Ca^2+^ were measured with qPCR in control CMs and CMs exposed to ischemia 6 h followed by 3 h of reperfusion. (**C** and **D**) Representative western blot results of comparison of SERCA2a, and RyR2 protein level in CMs exposed to ischemia and IR compared to control CMs; *n* = 3. (**E**) Representative immunofluorescence images of CMs stained with MitoSOX to detect the mitochondrial superoxide levels in control and after CMs exposed to ischemia 6 h followed by 2 h of reperfusion. Nuclei were stained with Hoechst 33,342 (blue), and the scale bar = 50 μm. (**F**) The values of MitoSOX fluorescence in mitochondria was expressed as mean ± SD. Comparisons between groups were made using the unpaired t-test. * for *p* < 0,05; ** for *p* < 0,01; *** for *p* < 0,001; *n* = 3

To determine the optimal times of reperfusion, we induced ischemia for 6 h followed by subsequent reperfusion for 2–4 h. After ischemia and respective reperfusion, we measured the changes in [Ca^2+^]_i_ with fluo-4 staining and mitochondrial superoxide was detected with MitoSOX. The significant accumulation level of [Ca^2+^]_i_ of both baseline (in resting) and peak (in contracting) was detected after 3 h of reperfusion (supplementary material Fig. S4A). In resting [Ca^2+^]_i_ indicated by fluo-4 fluorescence intensity in control CMs was 58.4 ± 10.24 and CMs exposed to IR was 92.13 ± 4.89 (*p* < 0.01) (Fig. 4A). Further, in contracting the level of [Ca^2+^]_i_ of control CMs was 99.76 ± 27.96 and CMs exposed to IR 173.86 ± 8.42 (*p* < 0.05) (Fig. 4A). To study the mechanism underlying Ca^2+^ transients in CMs during ischemia and IR, we next examined the gene expression and protein level of RyR2, which pumps Ca^2+^ from the endoplasmic reticulum to cytoplasm [29], and SERCA2a, which tries to dampen the Ca^2+^ influx [28]. Exposed CMs to IR significantly overexpressed RyR2 at gene (2.56 ± 0.8 vs. 1.0, *p* < 0.05) and protein level (2.6 ± 0.68 vs. 1.0, *p* < 0.01) in CMs in comparison with normoxia CMs (Fig. 4B-D). Also, SERCA2a significantly overexpressed at gene (1.53 ± 0.06 vs. 1.0, *p* < 0.001) and protein level (3.3 ± 1.24 vs. 1.0, *p* < 0.01) in CMs after exposed to IR in comparison with normoxia CMs (Fig. 4B-D). The CMs after 2 h of IR showed a significant increase in mitochondrial superoxide level, which plays a great role in the pathogenesis (supplementary material Fig. S4B). The mitochondrial superoxide detection was performed with stained cells with MitoSOX, which is specific for superoxide in mitochondria. MitoSOX fluorescence intensity was after 2 h of IR significantly higher in comparison to control (74.4 ± 15.5 vs. 108.0 ± 13.6, *p* < 0.05) (Fig. 4E, F). These data indicate that mitochondrial superoxide is involved and plays a role in IR injury.

### Intracellular ceramides play a significant role in IR injury

We hypothesized that intracellular ceramides have important implications in either mediating or causing injury and their inhibition improves the outcome of IR injury. To prove this hypothesis, we performed qPCR of ceramide synthase (*CerS*) genes. We found a significant increase in fold expression of *CerS2* (1.23 ± 0.02, *p* < 0.001) and *CerS4* (1.29 ± 0.13, *p* < 0.05) in CMs after IR compared to control CMs (Table 1). These genes encode the enzymes, that are involved in synthesis of different ceramide species, particularly long-chain and very long-chain ceramides [44]. Also, there was an upregulation in gene expression of *CerS1* and *CerS5* but no significance compared to control was detected (1.34 ± 0.28 and 1.66 ± 0.6 vs. 1.0 respectively) (Table 1). The expression of *SPTLC1*, which is involved in the *de novo* ceramide synthesis pathway, was not significantly changed after IR-CMs compared to control (0.96 ± 0.09 vs. 1.0, *p* = 0.41) (Table 1). Also, the gene of enzyme sphingomyelinase (*SMPD*), which is involved in the sphingomyelin pathway, was elevated in IR-CMs in comparison with control (1.53 ± 0.4 vs. 1.0, *p* < 0.05) (Table 1).

**Table 1:** Fold levels of mRNA (mean ± SD) of enzymes regulating ceramide synthesis in different pathways of their synthesis after IR injury

Gene	Control	Ischemia 6 h	Ischemia 6 h and reperfusion 16 h
*CerS1*	1.0	2.34 ± 1.27	1.34 ± 0.28
*CerS2*	1.0	1.19 ± 0.26	1.24 ± 0,02***
*CerS4*	1.0	0.66 ± 0.26	1.30 ± 0,13*
*CerS5*	1.0	0.88 ± 0.24	1.66 ± 0.60
*SMPD*	1.0	0.72 ± 0.09	1.53 ± 0.40*
*SPTLC1*	1.0	1.17 ± 0.28	0.96 ± 0.09

Comparisons between groups were made using the unpaired t-test. * for *p* < 0,05; ** for *p* < 0,01; *** for *p* < 0,001; *n* = 3

To confirm these data, we measured the ceramide content in CMs after ischemia and IR. We found a significant increase in total ceramide level (140.9 ± 40.6 *p* < 0.05) after IR in comparison to control (47.1 ± 20.5) (supplementary material table S3). Categorization of ceramides into individual ceramide species revealed that the concentration of long-chain ceramides C16:0 (55.9 ± 20.2 vs. 19.1 ± 12.9, *p* < 0.05), C18:0 (24.3 ± 7.4 vs. 8.5 ± 2.7, *p* < 0.05), and C18:1 (7.1 ± 1.8 vs. 0.9 ± 0.2, *p* < 0.01), as well as the very long-chain ceramides C22:0 (10.6 ± 3.5 vs. 3.1 ± 0.6, *p* < 0.05), and C24:1 (26.3 ± 7.1 vs. 9.5 ± 3.4, *p* < 0.05) were increased in CMs after IR in comparison to control (supplementary material table S3). No significant change was noticed in total ceramide level or ceramide species in CMs exposed to hypoxia (ischemia) compared to normoxia (control) CMs (supplementary material table S3). The results of an increase in *CerS* gene expression levels, a regulator of ceramide synthesis, and a significant increase in total ceramide levels after IR, confirm our assumption that intracellular ceramides have important implications in CMs-IR injury.

### Inhibition of ceramide level improves the outcome in CMs after exposed to IR

We hypothesize that inhibition of CerS during IR results in improved CMs viability and functions. To prove this concept, CMs were cultured during IR with fumonisin B1 (FB1) (50 μM). Interestingly, we found that the total level of ceramide and the levels of long-chain C16:0 (*p* = 0.19), C18:0 (*p* < 0.05), C18:1 (*p* < 0.05), and C20:0 (*p* < 0.01) as well as very long-chain C22:0 (*p* < 0.001) and C24:1 (*p* < 0.05) ceramide species were significantly lower in IR-CMs with FB1 inhibitor in comparison to IR-CMs alone (Table 2). Moreover, these cells showed that C18:1 (*p* < 0.05), C20:0 (*p* < 0.05), and C22:0 (*p* < 0.001) ceramide species were significantly lower in IR-CMs with FB1 in comparison to IR-CMs without FB1 (Table 2). This alteration in ceramide level confirmed our assumption that ceramide accumulation is involved in CMs-IR injury.

**Table 2:** Ceramide species determined in the cardiomyocytes (CMs) derived from UKJi005-A cell line (pmol/million cells)

N-acyl chain	Control	Ischemia 6 h followed by 16 h of reperfusion (IR)	
		IR	IR + FB1
C14:0	0.88 ± 0.34	2.12 ± 0.87	1.17 ± 1.27
C16:0	21.38 ± 9.76	50.97 ± 23.07*	23.09 ± 22.05
C16:1	0.84 ± 0.39	1.49 ± 0.49	1.43 ± 0.88
C18:0	21.38 ± 9.39	39.78 ± 10.67*	17.43 ± 7.22^#^
C18:1	2.16 ± 0.71	5.76 ± 1.34**	2.29 ± 2.11^#^
C20:0	2.74 ± 1.27	5.25 ± 0.89**	2.16 ± 0.93^##^
C20:1	0.34 ± 0.06	0.47 ± 0.11	0.27 ± 0.1^#^
C22:0	5.84 ± 1.77	11.61 ± 3.05***	4.19 ± 2.71^###^
C22:1	1.51 ± 0.44	2.59 ± 0.46*	1.54 ± 1.14
C24:0	4.06 ± 1.84	8.29 ± 4.48	2.54 ± 3.17
C24:1	11.71 ± 3.61	26.27 ± 8.31**	10.59 ± 82.64^#^
Total level	72.83 ± 291.5	154.6 ± 50.95*	66.7 ± 49.84^#^

**Fig. 5:**
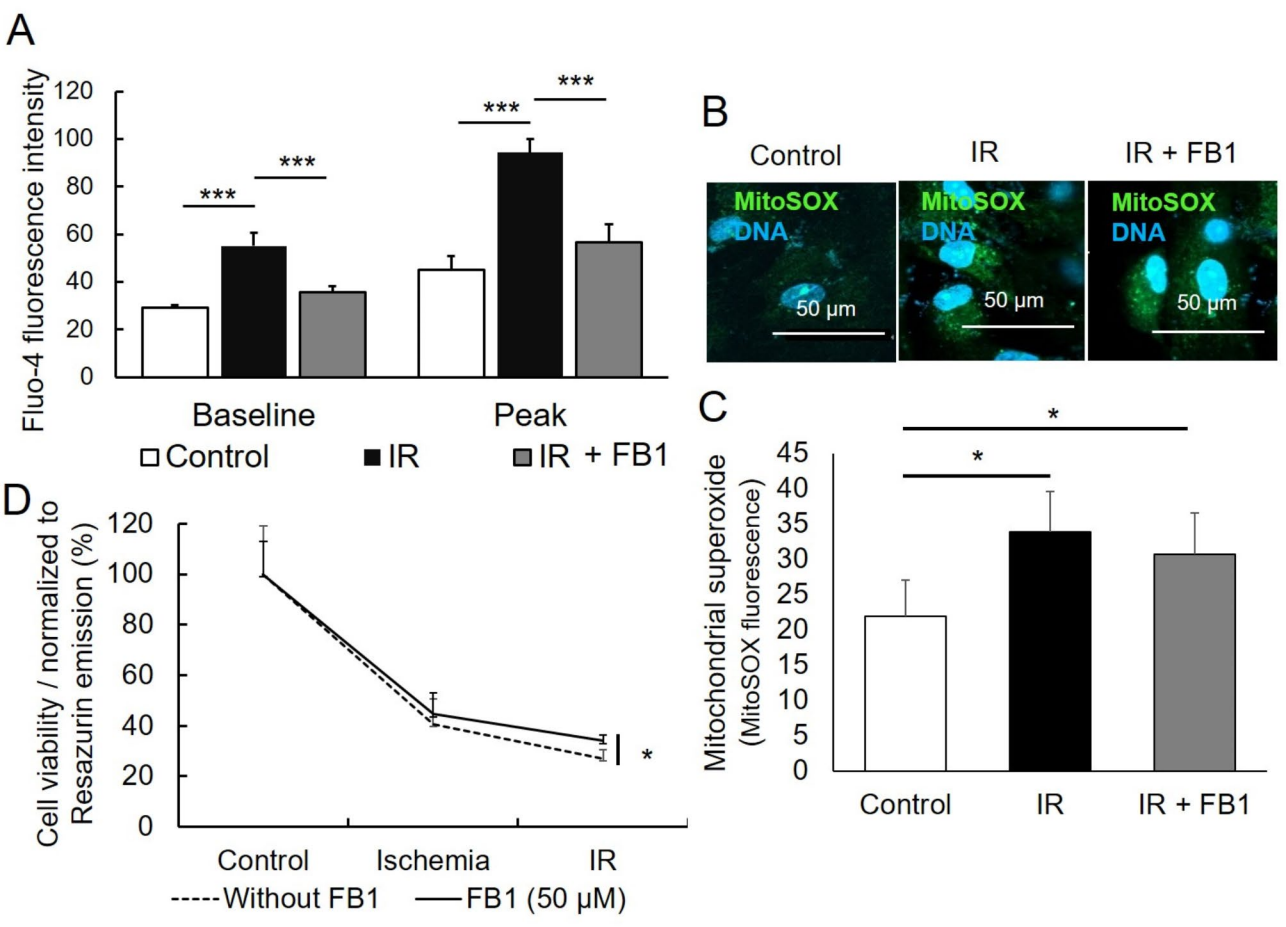
Inhibition of ceramide synthetase (Cer*S*) in cardiomyocytes (CMs) during ischemia/reperfusion (IR) injury leads to improved CM viability and functions. (**A**) Quantification of the [Ca^2+^]_i_ level in CMs indicated by changes of fluo-4 fluorescence intensity in the presence or absence of fumonisin B1 (FB1) (50 μM); *n* = 3. (**B**) Representative immunofluorescence images showing mitochondrial superoxide levels stained with MitoSOX in control CMs and IR-CMs using ceramide inhibitors FB1 (50 μM). Nuclei were stained with Hoechst 33,342 (blue), and the scale bar = 50 μm. (**C**) Quantification of the mitochondrial superoxide level in CMs indicated by changes of MitoSOX fluorescence intensity in the presence or absence of FB1 (50 μM); *n* = 3. (**D**) Assessment of cell viability assessed with PrestoBlue assay showed higher levels of significantly improved CM viability after incubation with FB1 (50 μM) (**E**). The values were expressed as mean ± SD Comparisons between groups were made using the one-way ANOVA, Multiple comparisons test, *p-value* consider, * for *p* < 0.05; ** for *p* < 0.01; *** for *p* < 0.001

Data are means ± SD; *n* = 6. One-way ANOVA, Multiple comparisons test, *p*-value consider, * for *p* < 0.05; ** for *p* < 0.01; *** for *p* < 0.001, and ^#^ for *p* < 0.05; ^##^ for *p* < 0.01; ^###^ for *p* < 0.001. * Compared to control or ^#^ compared to IR without inhibitor,

To see if inhibition of ceramide synthesis leads to lower levels of culprits such as [Ca^2+^]_i_ in terms of lower baseline and peak [Ca^2+^]_i_ levels, we incubated control and IR-CMs with and without FB1. As described above, the baseline and peak [Ca^2+^]_i_ was significantly higher in IR-CMs without FB1 inhibitor. The baseline of [Ca^2+^]_i_ in the control sample was 28.99 ± 1.2, and in reperfusion was significantly higher 54.99 ± 5.7 (*p* < 0.001) compared to control. Following FB1 (50 μM) treatment, CMs exposed to IR showed a significantly lower [Ca^2+^]_i_ (35.6 ± 2.5) (*p* < 0.001) (Fig. 5A). The peak of [Ca^2+^]_i_ in control was 45.1 ± 5.6 while reperfusion without FB1 inhibitor was significantly higher at 94.3 ± 5.7 (*p* < 0.001) and in reperfusion with FB1 56.5 ± 7.5 significantly lower (*p* < 0.001) (Fig. 5A). It was obvious that inhibition of the ceramides leads to less [Ca^2+^]_i_ load which could mean less damage to the CMs.

Mitochondrial superoxide is a very important culprit in all damages that are induced by ceramides [45]. In consistence with the results presented above, MitoSOX fluorescence intensity showed a higher level of mitochondrial superoxide in CMs exposed to IR without FB1 (33.83 ± 5.84) compared to control (21.98 ± 5.0) (Fig. 5B, C). However, it was found that FB1 (50 μM) does not significantly decrease the level of MitoSOX fluorescence intensity (30.66 ± 5.86) of IR-CMs compared to non-treated IR-CMs (Fig. 5B, C).

To know if the viability is improved, we evaluate cell viability by PrestoBlue assay. The results demonstrated a non-significant higher cell viability observed in CMs after 6 h of ischemia in the presence of FB1 in comparison to ischemia alone (44.6 ± 8.4% vs. 40.6 ± 10.1%, *p* = 0.62) (Fig. 5D). Importantly, after 6 h ischemia followed by 16 h reperfusion, the viability of CMs was significantly higher in the presence of FB1 in comparison to CMs exposed to IR without FB1 (34.0 ± 2.3% vs. 26.9 ± 3.7%, *p* < 0.05) (Fig. 5D).

It has been demonstrated that GATA4 and Nkx2.5 reduce IR injury through several of pathways, such as improving tissue healing, decreasing apoptosis, increasing cell survival, and improving heart function [46, 47]. Gene expression levels of GATA4 and Nkx2.5 were evaluated in CMs exposed to IR to gain a better understanding of the signaling mediated by ceramide (supplementary material Fig. S5). Only Nkx2.5 was found to be significantly reduced in CMs exposed to 6 h of ischemia (0.59 ± 0.16, *p* < 0.05) and after 16 h of CMs reperfusion (0.54 ± 0.14, *p* < 0.01) (supplementary material Fig. S5).

The Nkx2.5 gene expression level of IR-CMs was unaffected by FB1 (50 μM) incubation (0.63 ± 0.19, *p* < 0.05) (supplementary material Fig. S5). According to the findings, ischemia or IR’s effect on Nkx2.5 inhibition was not significantly mitigated by ceramide inhibition, indicating that the Nkx2.5 pathway acts upstream of the ceramide pathway.

Together, the reduction in ceramide levels may help, but not completely restore, the results of IR in CMs, supporting our hypothesis that the elevated ceramide level after IR exposure plays a role in CMs viability and functions.

### Mitochondrial function in CMs could be protected by lowering the ceramide overload associated with IR injury

**Fig. 6:**
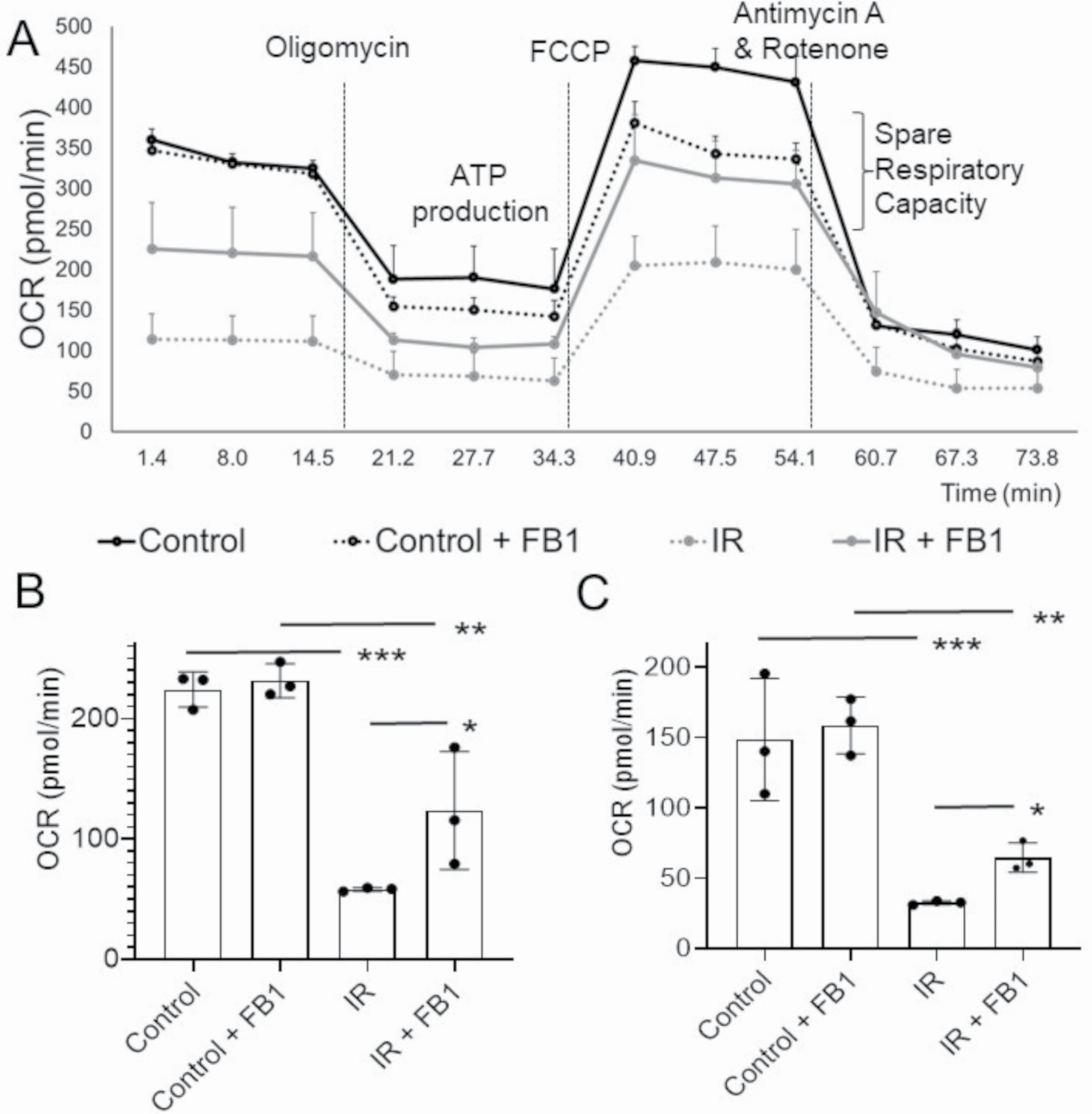
Relationship between the effects of IR-induced ceramide signaling and mitochondrial respiration in cardiomyocytes (CMs). (**A**) FA β-oxidation in mitochondrial respiration was determined by calculating the oxygen consumption rate (OCR) using a Seahorse XF96 analyzer. (**B**) Mitochondrial respiration at a basal level compared to control. (**C**) ATP production in control CMs and IR samples using ceramide inhibitors fumonisin B1 (FB1) (50 μM). Comparisons between groups were made using the one-way ANOVA, Multiple comparisons test, *p*-value consider, * for *p* < 0.05; ** for *p* < 0.01; *** for *p* < 0.001

Here, we demonstrated the tight relationship between the effects of IR-induced ceramide signaling and mitochondrial function (Fig. 6A). We show a significant decrease (*p* < 0.001) in mitochondrial function at the basal level (58.09 ± 1.55) compared to control (224.15 ± 14.53) (Fig. 6B) and FA ß-oxidation by calculating the oxygen consumption rate (OCR) using a Seahorse XF96 analyzer, suggesting a significant reduction (*p* < 0.001) of ATP production in IR-CMs (32.54 ± 1.4) compared to control (148.66 ± 43.42) (Fig. 6C). Interestingly, incubation of CMs with FB1 (50 μM) significantly improved mitochondrial function (*p* < 0.05) as well as ATP production (64.71 ± 10.44) compared to non-treated IR-CMs (32.54 ± 1.4) (Fig. 6A-C).

### The overexpression of the CerS2 gene in the CMs of transgenic α-MHC-CerS2 mice had an impact on the heart tissue

**Fig. 7:**
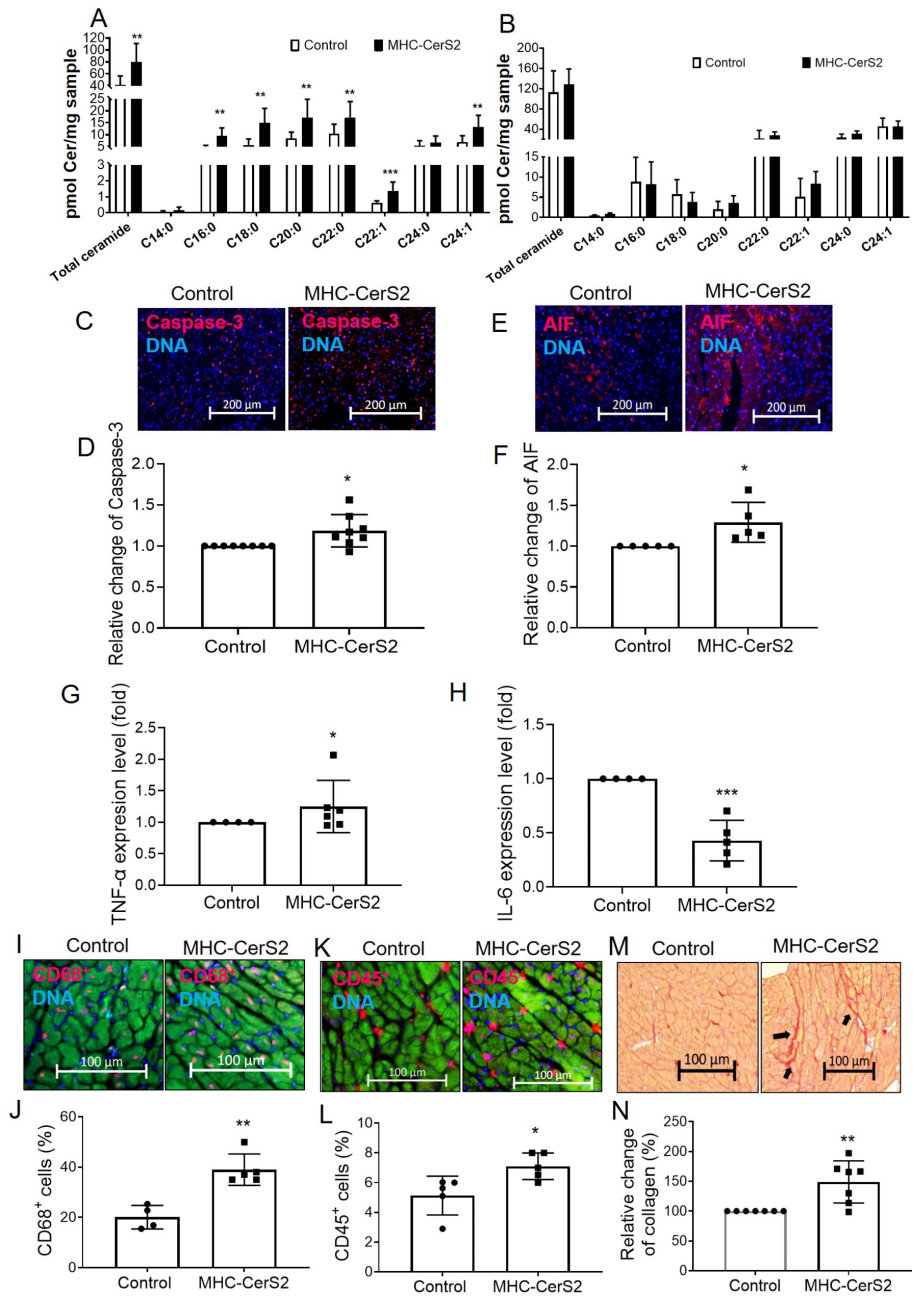
The overexpression of the CerS2 gene in the CMs of α-MHC-CerS2 mice had an impact on the heart tissue. (**A**) Heart tissues had higher levels of total ceramide level and long-chain, as well as very long-chain ceramide species in MHC-Cers2 mice (*n = 10*) compared with control mice (*n = 8*). (**B**) No changes in the ceramide level in the serum of MHC-CerS2 mice (*n = 10*) compared with control mice (*n = 8*). (**C**) Representative immunofluorescence images for apoptosis marker caspase-3 in heart sections. (**D**) Quantification of the fold change of positive cells of caspase-3 (red) to nuclei stained with Hoechst 33,342 (blue) showing the higher level of apoptosis caspase-3 in heart tissues of MHC-CerS2 mice. (**E**) Representative immunofluorescence images of apoptosis inducing factor (AIF) in heart sections, which is a mitochondrial oxidoreductase that contributes to cell death programs. Quantification of the fold changes positive cells of AIF (red) to nuclei stained with Hoechst 33,342 (blue) showing the higher level of apoptosis in heart tissues of MHC-CerS2 mice. (**G** and **H**) Gene expression related to the inflammatory response in the heart, such as TNF-α and IL-6 were altered in heart tissues of MHC-CerS2 mice. (**I**) Representative immunofluorescence images of CD68^+^ cells in heart sections. (**J**) Quantification of the change positive CD68^+^ cells (red) to nuclei stained with Hoechst 33,342 (blue) showing the higher level of inflammatory cell infiltration in heart tissues of MHC-CerS2 mice. (**K**) Representative immunofluorescence images of CD68^+^ cells. (**J**) Quantification of the change positive CD45^+^ cells (red) to nuclei stained with Hoechst 33,342 (blue) showing the higher level of inflammatory cells infiltration in heart tissues of MHC-CerS2 mice (**M**) Representative images of the Sirius Red staining in heart sections. (**N**) Quantification of the percentage of the cardiac collagen I and III deposition in heart sections showing the higher collagen in heart tissues of MHC-CerS2 mice. Comparisons between groups were made using the unpaired t-test. * for *p* < 0,05; ** for *p* < 0,01; *** for *p* < 0,001

To confirm the initial hypothesis that long-chain and very long-chain ceramides contribute to heart disease and to examine the impact of elevated ceramide levels in the heart, we used a transgenic mouse model, where the Cers2 gene is overexpressed in the CMs of MHC-CerS2 mice. We examined whether mice’s heart tissue and serum ceramide levels were affected by the overexpression of CerS2 in CMs. We found that only the heart tissues had higher levels (*p* < 0.01) of total ceramide in MHC-CerS2 mice (80.02 ± 30.71, *n* = 10) compared with control mice (49.33 ± 8.66, *n* = 8) (Fig. 7A). However, no changes (*p* = 0.85) in the ceramide level in serum of MHC-CerS2 mice (136.6 ± 58.98, *n* = 10) compared with control mice (128.1 ± 22.47, *n* = 8) (Fig. 7B). Among all ceramide species analyzed, we found that the level of long-chain (C16:0 and C18:0) and very long-chain (C20:0, C22:0, C20:1, and C24:1) ceramide species were significantly higher in heart tissues of MHC-CerS2 mice when administered with doxycycline (0.545 mg/g) for one month compared with control mice (Fig. 7A, B). Furthermore, higher levels of apoptosis caspase-3 (1.19 ± 0.15, *p* < 0.05) (Fig. 7C, D) and apoptosis inducing factor (AIF) (1.29 ± 0.24, *p* < 0.05) were detected in heart tissues of MHC-CerS2 mice (Fig. 7E, F). This finding confirmed that CerS2-overexpression in CMs resulted in an increase in the long-chain and very long-chain of ceramides, which leads to increased apoptosis in heart tissues.

Next, we analyzed whether an increase ceramide levels in CMs of MHC-CerS2 mice could induce the inflammatory response in the heart. Here we show that increased ceramide in CMs has a significant effect on proinflammatory cytokines. Gene expression analysis demonstrated upregulation of TNF-α mRNA (1.46 ± 0.21, *p* < 0.05) (Fig. 7G) and downregulation of IL-6 mRNA (0.42 ± 0.18, *p* < 0.001) (Fig. 7H) in heart tissues of MHC-CerS2 mice. Furthermore, a significant increase in interstitial inflammatory cells infiltration, such as CD68^+^ cells (38.98 ± 3.77, *p* < 0.01) (Fig. 7I, J) and CD45^+^ (7.16 ± 0.7, *p* < 0.05) (Fig. 7K, L) cells in heart tissues sections of MHC-CerS2 mice. Also, to evaluate cardiac collagen deposition, collagen fibers were detected by Sirius Red staining in heart sections, overexpressed CerS2 in CMs induced a significant increase in collagen (204.02 ± 36.9, *p* < 0.05) in cardiac tissues of MHC-CerS2 mice compared with control mice (Fig. 7M, N). In conclusion, the data provided here suggests that elevated ceramide levels in CMs play an essential role in the inflammatory response, fibrosis, and apoptosis of the heart tissues.

## Discussion

Ischemia/reperfusion (IR) injury has been addressed previously and investigated at the level of in vitro and in vivo models [48, 49]. However, the evidence remains insufficient to recommend an effective agent to reduce the risk of reperfusion injury [50]. Part of this failure to translate therapies can be attributed to an incomplete understanding of the mechanisms under myocardial reperfusion injury as well as the difference between preclinical animal models and clinical scenarios in patients [51, 52]. For that, it is clear more studies need to be done on human-cell-based cardiomyocytes (CMs) as in vitro models to minimize IR injury. To investigate the role of ceramides in IR injury, we generated hiPSC.FS.2 (UKJi005-A) that can differentiate to human CMs. To mimic the well-known transient changes in cardiac IR injury in vivo, we use matured CMs derived from UKJi005-A cell line to establish an in vitro IR injury model. Mature hiPSC-derived CMs produce about 60–70% of the total ATP via fatty acid ß-oxidation. Utilizing immature CMs produced from iPSC may influence the result of the IR injury cell model, since these cells primarily rely on glycolysis for ATP generation [38]. Increased glycolysis allows the shuttling of glycolytic intermediates to hexosamine biosynthesis pathway, since it introduces the O-GlcNAcylation modification in protein sequences [53]. Elevated O-GlcNAc levels have been linked to heart failure, myocardial remodeling, and pressure-overload-induced hypertrophy in the cardiovascular system, all of which enhance mortality during ischemia [53]. Furthermore, the T-tubule network is absent from immature hiPSC-derived CMs [54]. This leads to poor Ca^2+^ channel and RyR co-localization, resulting in the kinetics of the Ca^2+^ transients to be unsynchronized and slowing down [55, 56]. To establish an in vitro IR injury model, hiPSC-derived CMs were exposed to hypoxia/reoxygenation based on the in vitro model of Wei et al. [39] with slightly modified exposure times, which resembles IR in vivo. In agreement with others [57, 58], we found the known characteristics of IR injury in our model after exposure to hypoxia/reoxygenation. According to the results, hypoxia (ischemia) as well as reoxygenation (reperfusion) induced mitochondrial superoxide accumulation and intracellular Ca^2+^ [Ca^2+^]_i_ overload as possible factors that could be responsible for injury in reperfusion [59]. Further, we proved that apoptosis could also be the cause of this additional death of CMs. In this study, these criteria were met with 6 h of ischemia time followed by 16 h of reperfusion. With this exposure time CM viability was significantly lower after IR in comparison to ischemia alone. These findings are in line with previous studies regarding the lower cell viability for many in vitro models of IR injury that exist with different exposure times of ischemia (2–5 h) [39, 57, 60–62] and reperfusion (4–16 h) [60–62].

Heart failure with IR injury is significantly influenced by abnormal [Ca^2+^]_i_ homeostasis [63–65]. The cytosol had a significantly more Ca^2+^ overload at both baseline and peak levels, which is consistent with earlier findings in other models [66–68]. It is already known that Ca^2+^ plays a role in mediating many processes, which lead to apoptosis [69]. Even in reperfusion injury, increased Ca^2+^ leads to mitochondrial dysfunction and hypercontracture state and finally to more CMs death [69]. We could confirm that the interplay between increased *RyR2* and *SERCA2a* mRNA expression leads to more Ca^2+^ in the cytoplasm and hypercontracture, resulting in more cell damage and apoptosis [29]. With net higher levels of [Ca^2+^]_i_, we could hypothesize that during IR, SERCA2a even though overexpressed, cannot outweigh the effects of *RyR2*. We can conclude that *SERCA2a* and its over-activation and *RyR2* and its deactivation during IR remain interesting targets for improving IR injury.

Changes in the level of *CerS* and ceramide level in human CMs were also investigated. During ischemia, there were no significant changes in the *CerS* genes and ceramide level. However, after reperfusion, we observed higher expression of *CerS* genes and intracellular levels of ceramides, which confirmed the role played by ceramides in causing/mediating the development of IR injury in human CMs.

The contribution of intracellular ceramide level on [Ca^2+^]_i_ at IR injury has been evaluated in human CMs. To address this issue, the present results clearly showed an elevation of ceramide levels contributed to an increase in the [Ca^2+^]_i_ in CMs exposed to IR directly, which could lead to proving the hypothesis that they could play a role in causing/mediating injury in IR [70, 71]. To achieve a better understanding of the role of ceramide on [Ca^2+^]_i_ in IR injury, we measured the change in [Ca^2+^]_i_ of CMs in the presence of fumonisin B1 (FB1), which is an inhibitor of CerS. The results obtained with the presence of FB1 during IR support our hypothesis that ceramide regulates [Ca^2+^]_i_ during IR and its inhibition improves [Ca^2+^]_i_ alterations and increases CMs viability during IR injury. Also, we demonstrate that FB1 has a role in reducing IR injury in human CMs and viability improvement. These findings support several other research that indicates modulating the ceramide-generating pathway may be a promising treatment to overcome IR injury [70, 72].

It has been demonstrated that WT1, GATA4, and Nkx2.5 reduce IR injury through a number of pathways, such as improving tissue healing, decreasing apoptosis, increasing cell survival, and improving heart function [46, 47, 73]. Here we evaluated the gene expression levels of *GATA4* and *Nkx2.5* in CMs exposed to IR to gain a better understanding of the signaling mediated by ceramide. Only *Nkx2.5* was found to be significantly reduced in CMs exposed to ischemia and IR. Also, the Nkx2.5 gene expression level of IR-CMs was unaffected by FB1 incubation. According to the findings, ischemia or IR’s effect on Nkx2.5 inhibition was not significantly mitigated by ceramide inhibition, indicating that the Nkx2.5 pathway acts upstream of the ceramide pathway.

Here, we demonstrate a substantial decline in mitochondrial activity, pointing to a marked decrease in ATP synthesis in IR-CMs relative to control CMs. Furthermore, compared to untreated IR-CMs, incubation of CMs with FB1 markedly enhanced mitochondrial activity and ATP generation. This suggests that elevated ceramide levels raise [Ca^2+^]_i_, just as Ca^2+^ overloading during IR damage causes CMs to develop mitochondrial dysfunction. Hence, protecting mitochondria from ceramide overload associated with IR may maintain mitochondrial function and minimize cardiac damage and subsequent failure. The process of oxidative phosphorylation in mitochondria produces ATP, which is necessary for the SR to control contraction and other organelles such as interfibrillar mitochondria, which are found between myofibrils and closely associated with the SR, and subsarcolemmal mitochondria, which are situated beneath the sarcolemma.

One limitation to our study that is important to acknowledge. The results of ischemia are not measured in a given ischemia. Since the experiments after incubation under ischemia measurements were made in normal O_2_ concentration, so a little reperfusion was present. For this reason, we did not focus on our ischemia results. New experiments in strict conditions are needed to establish the role of ceramides in ischemia alone.

Ceramide inhibition, especially through CerS1, which is primarily expressed in skeletal muscle and neuronal tissue, has worsened age-related weakness of muscles by reducing myogenesis and increasing fibrosis and inflammation [74]. This affects the condition of muscles and maybe the brain because compensatory mechanisms raise harmful long-chain ceramides [74]. However, to avoid compensatory mechanisms’ long-term effects of CerS2 inhibition, which is highly expressed in the heart and liver as well as nervous tissue [44, 75]. Further, to confirm the fundamental theory that ceramide contributes to cardiac disease in vivo, we created a transgenic mouse model in which the CerS2 gene was locally and temporarily overexpressed in the heart of MHC-CerS2 mice after doxycycline administration. This allowed us to investigate the effects of long-chain and very long-chain ceramide in CMs. When doxycycline was given to MHC-CerS2 animals for a month, our findings demonstrated that the level of long-chain and very long-chain ceramide species was significantly higher in the heart tissue but not in their serum in comparison to control mice. Interestingly, we found consistency between the type of elevated ceramide species in MHC-CerS2 mice’s heart tissues and type of ceramide species in CMs derived from hiPSC after IR. We further show that high levels of ceramides in MHC-CerS2 mice’s heart tissue significantly increased collagen deposition, proinflammatory cytokines, interstitial inflammatory cell infiltration, and apoptosis in vivo. Further, It has also been demonstrated that overexpression of CerS2 in CMs results in lipid overload, oxidative stress, and mitochondrial dysfunction, which in turn causes apoptosis [19, 76]. Taken together, these results support our hypothesis that ceramide plays a role in CMs damage. Additionally, the findings support the idea that attenuated ceramide can help with IR damage to the heart.

## Conclusions

To conclude, we established an in vitro model from hiPSC-derived CMs, permitting investigation of the molecular mechanisms of IR injury with the potential to introduce a new target. As such we anticipate that reducing the elevated levels of ceramides in reperfusion injury will significantly improve mitochondrial function and viability of CMs by lowering [Ca^2+^]_i_ levels, which may provide an opportunity to identify a new regulator of the IR injury. Furthermore, in vivo experiments demonstrate that elevated levels of ceramides significantly increase interstitial inflammatory cell infiltration, collagen deposition, proinflammatory cytokines, and apoptosis in the heart of mice. Therefore, our study provides an outline for additional research investigating the mechanisms linking ceramides and IR injury in human CMs and proving that ceramide inhibitors could be important therapeutic targets.

## Electronic supplementary material

Below is the link to the electronic supplementary material.

Supplementary Material 1

Supplementary Material 2

## Data Availability

All data are available in the main text or the supplementary materials.

## Declarations

### Artificial intelligence (AI)

The authors declare that they have not use AI-generated work in this manuscript.

## Abbreviations

CerS: Ceramide synthase
cLSM: Confocal laser scanning microscope
CMs: Cardiomyocytes
DMSO: Dimethyl sulfoxide
FB1: Fumonisin B1
FDA: Fluorescein diacetate
hiPSC: Human induced pluripotent stem cell
IR: Ischemia/reperfusion
PI: Propidium iodide
ROS: Reactive oxygen species
RYR2: Ryanodine receptor 2
SERCA2a: Sarcoendoplasmic reticulum calcium ATPase 2a
SPTLC: Serine-palmitoyltransferase long chain base subunit
