# Supplementary material for: Inhibition of ceramide synthesis improves the outcome of ischemia/reperfusion injury in cardiomyocytes derived from human induced pluripotent stem cell
Source: Stem Cell Res Ther. 2025 Apr 18;16:190. doi: 10.1186/s13287-025-04340-3 (PMC12008854; doi:10.1186/s13287-025-04340-3)
Supplement: Supplementary file 1 — Supplementary Material 1 [file 13287_2025_4340_MOESM1_ESM.docx]

**Supplementary tables and figures**

**Inhibition of ceramide synthesis improves the outcome of ischemia/reperfusion injury in cardiomyocytes derived from human induced pluripotent stem cell**

Pellumb Haxhikadrija^1^, Jasmine M.F. Wu^1^, Sascha Hübner^1^, Katja Grün^1^, Tom Kretzschmar^1^, Tina Müller^2^, Markus H. Gräler^2^, Claudia Backsch^3^, Anja Weise^4^, Elisabeth Klein^4^, P. Christian Schulze^1^*, Mohamed M. Bekhite^1^*

^1^Department of Internal Medicine I, Division of Cardiology, University Hospital Jena, Friedrich-Schiller-University, Jena, Germany

^2^Department of Anesthesiology and Intensive Care Medicine and Center for Molecular Biomedicine (CMB), University Hospital Jena, Friedrich-Schiller-University, Jena, Germany

^3^Department of Gynecology and Reproductive Medicine, Jena University Hospital Jena, Friedrich-Schiller-University, Jena, Germany

^4^Institute of Human Genetics, University Hospital Jena, Friedrich-Schiller-University, Jena, Germany

* Contributed equally to this manuscript

Corresponding author:

Dr. Mohamed M. Bekhite

University Hospital Jena

Department of Internal Medicine I,

Division of Cardiology

Am Klinikum 1

07747 Jena, Germany

[Mohamed.el_Saied@med.uni-jena.de](mailto:Mohamed.el_Saied@med.uni-jena.de)

Phone: 0049-3641-9325813

Fax: 0049-3641-9325812

**Methods**

**Animal models for in vivo study**

1. MHC-Tet3G transgenic mouse line

This mouse line expresses the doxycycline-inducible transactivator, which is regulated by the expression of the specific myocardial marker gene α-MHC. This mouse line was necessary to mate with the pTRE3G-CerS2 mice. The resulting offspring have the specific myocardial transactivator and the corresponding gene, the expression of which can be switched on as required by administering doxycycline. The mice did not show a burdened phenotype.

2-pTRE3G-CerS2 transgenic mouse line

This mouse line expresses the tetracycline response element (TRE), which regulates the expression of CerS2. Mating with the MHC-Tet3G mouse line produces offspring that express the α-MHC-controlled transactivator, the TRE and CerS2. The administration of doxycycline can regulate the specific overexpression of CerS2 locally and temporarily in the heart. The administration of doxycycline leads to binding and activation of the transactivator. This then binds to the tetracycline response element (TRE), which enables the transcription of the downstream gene CerS2. The mice did not show a burdened phenotype.

3-MHC-CerS2 transgenic mouse line

This mouse line was created by mating the mice MHC-Tet3G and pTRE3G-CerS2. It contains both the α-MHC-controlled transactivator and the TRE-controlled CerS2 gene. Cardiac-specific CerS2 expression is activated after doxycycline administration. Animals older than 8 weeks, regardless of gender, were used for the experiments.

4-C57BL/6J wild-type mice

Animals older than 8 weeks, regardless of gender, were used as a control group for the experiments.

To check the correct and necessary genotype, genotyping was carried out using PCR and subsequent gel electrophoresis. The following primer pairswere used for this.

|  | **Forward primer** | **Reverse primer** |
| --- | --- | --- |
| **α-MHC-Tet3G** | GTCGACACCATGTCTAGACTGG | TGACACAGGAACGCGAGC |
| **pTRE3G-CerS2** | CCATGATGGGAGTTCTACAGCTGC | TGACGCGTGATATCCGGC |

MHC-CerS2 double positive mice were determined by expression of both α-MHC-Tet3G and pTRE3G-CerS2.

**Doxycycline administration via food**

Mice older than 8 weeks, regardless of gender, were used. The literature and previously unpublished results have shown that administration of 2 mg doxycycline/mouse/day activates target gene expression [1]. To ensure sufficient intake of doxycycline for gene activation, the mice are fed doxycycline supplementary food at a concentration of 0.545 mg/g. Assuming that the average intake via food is 4 g/mouse/day, feeding doxycycline food at this concentration leads to an intake of doxycycline of 2.18 mg/mouse/day for one month. We also avoided intraperitoneal injections, as we did not want to subject the mice to unnecessary stress.

| **Table S1**. List of antibodies used in this study | | | |
| --- | --- | --- | --- |
| Antibody | | Host | Supplier |
| Anti-ßIII-Tubulin | | Rabbit | Abcam |
| Anti-α-actinin | | Mouse | Merck |
| Anti-MYL2 (MLC2v) | | Rabbit | Abcam |
| Anti-α-smooth muscle actin | | Mouse | Abcam |
| Anti-α 1 fetoprotein | | Rabbit | Abcam |
| Anti-SOX2 | | Mouse | Abcam |
| Anti-Nanog | | Rabbit | Abcam |
| Anti-TRA-1-60 | | Rabbit | Abcam |
| Anti-OCT4 | | rabbit | Abcam |
| Anti-Caspase-3 | | Rabbit | Cell Signaling Technology |
| Anti-AIF | | Rabbit | Abcam |
| Anti-mouse CD68 | | Rat | Biolegend |
| Anti-mouse CD45 | | Rat | Biolegend |
| Anti-Mouse IgG CY2 | | Goat | Abcam |
| Anti-Mouse IgG CY5 | | Goat | Abcam |
| Anti-Rabbit IgG CY3 | | Goat | Abcam |
| Anti-Rabbit IgG CY5 | | Goat | Abcam |
| Anti-Rat IgG Cy5 | | Donkey | Abcam |
| Anti-SERCA2 ATPase | | Rabbit | Abcam |
| Anti-Ryanodine receptor 2/RYR-2 | | Rabbit | Abcam |
| Anti-Bcl-2 | | Rabbit | Abcam |
| Anti-Bax | | Rabbit | Abcam |
| Anti-Vinculin | | Rabbit | Abcam |
| Anti- Rabbit HRP | | Goat | Abcam |
| Abcam, Cambridge, UK | | |  |
| Merck, Darmstadt, Germany  Cell Signaling Technology, Frankfurt, Germany  Biolegend, London, UK | | |  |

| **Table S2.** | | | |
| --- | --- | --- | --- |
|  | | | |
| 1. **Human primers used in this study** | | | |
| Primer |  | Sequence | Size (bp) |
| *OCT4* | Sense | 5'-CTGAGGGCGAAGCAGGAG-3' | 241 |
|  | Antisense | 5'-AATAGAACCCCCAGGGTGAG-3' |  |
| *SOX2* | Sense | 5'-ACACCAATCCCATCCACACT | 224 |
|  | Antisense | 5'-GCAAACTTCCTGCAAAGCTC |  |
| *NANOG* | Sense | 5'-GATTTGTGGGCCTGAAGAAA-3' | 155 |
|  | Antisense | 5'-AAGTGGGTTGTTTGCCTTTG-3' |  |
| *α-actinin* | Sense | 5'-GCTATATCCCGGCCATAAAC-3' | 99 |
|  | Antisense | 5'-TACCCACACCCATCCTAAA-3' |  |
| *MLC2a* | Sense | 5'-CAGGCCCAAGGTGGTTCTT-3' | 141 |
|  | Antisense | 5'-CCATCACGATTCTGGTCGATA-3' |  |
| *MLC2v* | Sense | 5'-CCTTGGGCGAGTGAACGT-3' | 120 |
|  | Antisense | 5'-GGGTCCGCTCCCTTAAGTTT-3' |  |
| *HCN4* | Sense | 5'-GTCTTTGTTTGGGGCAAGAG-3' | 113 |
|  | Antisense | 5'-GATTGGATGGCAGTTTGGAG-3' |  |
| *CerS1* | Sense | 5'-CAATGTGGGCATCCTTGT-3' | 89 |
|  | Antisense | 5'-CGCGGGACTTGAAGTAAAT-3' |  |
| *CerS2* | Sense | 5'-GGCCGTCATTGTGGATAAA-3' | 93 |
|  | Antisense | 5'-TACCAATACTGGGAAGGGATAG-3' |  |
| *CerS4* | Sense | 5'-CTTCTTCGGCTACTACTTCTTC-3' | 117 |
|  | Antisense | 5'-TCCATCTGGCCCTTCTT-3' |  |
| *CerS5* | Sense | 5'-CCTACAGCTTCTGCATGTC-3' | 93 |
|  | Antisense | 5'-CTGCGATCATCCTTCGATAC-3' |  |
| *CerS6* | Sense | 5'-GGCTAAGCACAGGTCATAAG-3' | 95 |
|  | Antisense | 5'-CCCTATGCTCAACATCCATAG-3' |  |
| *SPTLC1* | Sense | 5'-CCAGTGCTATTCCTGCTTAC-3' | 93 |
|  | Antisense | 5'-GGGATGCCTGTAATCCTTTC-3' |  |
| *SMPD1* | Sense | 5'-GTTCAACCAGGCAAGATCA-3' | 96 |
|  | Antisense | 5'-CTAAACCAGCTCCAGGAAAG-3' |  |
| *Caspase-3* | Sense | 5'-GAGCTGCCTGTAACTTG-3' | 214 |
|  | Antisense | 5'-ACCTTTAGAACATTTCCACT-3' |  |
| *Bcl-2* | Sense | 5'-TTGGATCAGGGAGTTGGAAG-3' | 295 |
|  | Antisense | 5'-TGTCCCTACCAACCAGAAGG-3' |  |
| *Bax* | Sense | 5'-GGACGAACTGGACAGTAACATGG-3' | 150 |
|  | Antisense | 5'-GCAAAGTAGAAAAGGGCGACAAC-3' |  |
| *GATA4* | Sense | 5'-GACAATCTGGTTAGGGGAAGC-3' | 105 |
|  | Antisense | 5'-GAGAGATGCAGTGTGCTCGT-3' |  |
| *NKX2.5* | Sense | 5'-ACCCTGAGTCCCCTGGATTT-3' | 125 |
|  | Antisense | 5'-TCACTCATTGCACGCTGCAT-3' |  |
| *GAPDH* | Sense | 5'-GTGGACCTGACCTGCCGTCT-3' | 153 |
|  | Antisense | 5'-GGAGGAGTGGGTGTCGCTGT-3' |  |
| *SeV* | Sense | 5'-GGATCACTAGGTGATATCGAGC-3' | 181 |
|  | Antisense | 5'-ACCAGACAAGAGTTTAAGAGATATGTATC-3' |  |
| *SeV-KOS* | Sense | 5'-ATGCACCGCTACGACGTGAGCGC-3' | 528 |
|  | Antisense | 5'-ACCTTGACAATCCTGATGTGG-3' |  |
| *SeV-KLF4* | Sense | 5'-TTCCTGCATGCCAGAGGAGCCC-3' | 410 |
|  | Antisense | 5'-AATGTATCGAAGGTGCTCAA-3' |  |
| *SeV-c-MYC* | Sense | 5'-TAACTGACTAGCAGGCTTGTCG-3' | 532 |
|  | Antisense | 5'-TCCACATACAGTCCTGGATGATGATG-3' |  |
| 1. **Mouse primers used in this study** | | | |
| *CerS2* | Sense | 5'-GAGCCCACTCTGCCGTGACAA-3' | 193 |
|  | Antisense | 5'-GAGCCCACTCTGCCGTGACAA-3' |  |
| *IL-6* | Sense | 5'-TCTGGGAAATCGTGGAAATGAGA-3' | 180 |
|  | Antisense | 5'-ACTCCAGACCAGAGGAAA-3' |  |
| *TNF-α* | Sense | 5'-AACCTCCTCTCTGCCGTCAA-3' | 141 |
|  | Antisense | 5'-GCAGATTGACCTCAGCGCT-3' |  |
| *GAPDH* | Sense | 5'-TGGCCTCCAAGGAGTAAGAA-3' | 201 |
|  | Antisense | 5'-TTGTGGGTGCAGCGAACTTTA-3' |  |

- Table S3. Intracellular ceramides in hiPSC-FS.2 (UKJi005-A) derived cardiomyocytes (CMs) during ischemia/reperfusion (IR) injury. Ceramide levels (pmol/million cells) of long-chain and very long-chain ceramide species were measured with mass spectrometry in cardiomyocytes after ischemia and IR compared to control.

| N-acyl chain | Control | Ischemia 6 hrs | Ischemia 6 h + Reperfusion 16 hrs |
| --- | --- | --- | --- |
| C14:0 | 0,32±0,18 | 0,48±0,30 | 1,06±0,34 |
| C16:0 | 19,13±12,94 | 15,53±5,87 | 55,90±20,15*^#^ |
| C16:1 | 0,41±0,09 | 0,58±0,24 | 1,05±0,35 |
| C18:0 | 8,47±2,72 | 12,28±6,81 | 24,33±7,37*^#^ |
| C18:1 | 0,86±0,11 | 1,22±0,85 | 7,10±1,80*^#^ |
| C20:0 | 1,46±0,72 | 1,53±0,82 | 3,22±0,63 |
| C20:1 | 0,11±0,01 | 0,12±0,10 | 0,31±0,07 |
| C22:0 | 3,01±0,61 | 3,25±1,86 | 10,64±3,50*^#^ |
| C22:1 | 0,66±0,19 | 0,71±0,42 | 1,44±0,30 |
| C24:0 | 3,12±0,22 | 2,60±1,21 | 9,56±4,48*^#^ |
| C24:1 | 9,57±3,39 | 8,64±3,14 | 26,26±7,14*^#^ |
| Total level | 47,13±20,47 | 46,95±21,21 | 140,87±40,56*^#^ |

* Compared to control, # compared to Ischemia 6 hrs

Data are means ± SD; *n=3*. One-way ANOVA, Multiple comparisons test, p value consider, * for *p*<0.05; ** for *p*<0.01; *** for *p*<0.001, and ^#^ for *p*<0.05; ^##^ for *p*<0.01; ^###^ for *p*<0.001. * Compared to control or ^#^ compared to ischemia.

**Supplementary Figures**


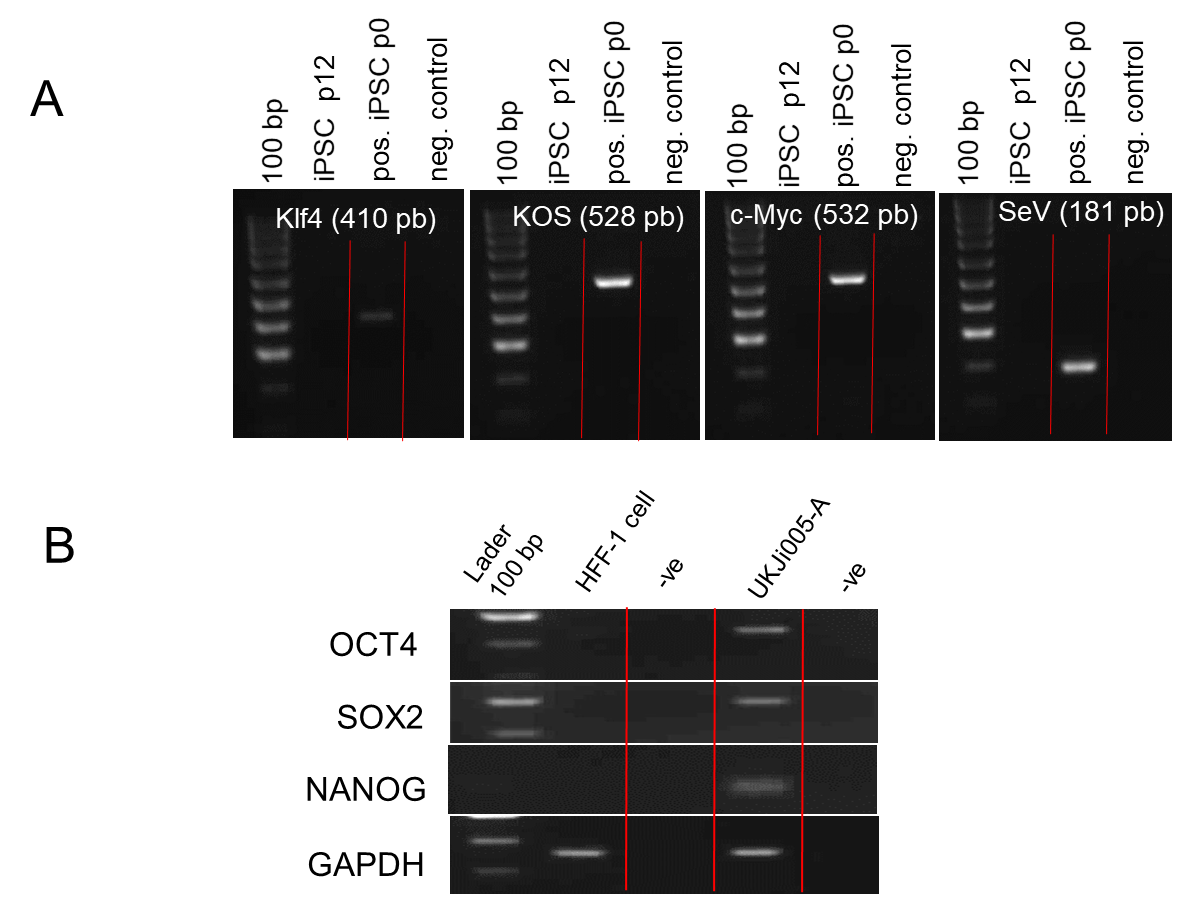


**Fig. S1:** Reverse transcription-polymerase chain reaction (RT-PCR) analysis showed (A) Absence of Sendai virus particles in hiPSC-FS.2 (UKJi005-A) was analysed RT-PCR for reprogramming vectors in positive control iPSC (p0) and iPSC (p12). (B) Enhanced endogenous pluripotent genes expression *Oct4, Nanog*, and *Sox2* in UKJi005-A colony compared with HFF.1 cell. The housekeeping gene glyceraldehyde-3-phosphate dehydrogenase (GAPDH) was used as an internal reference.


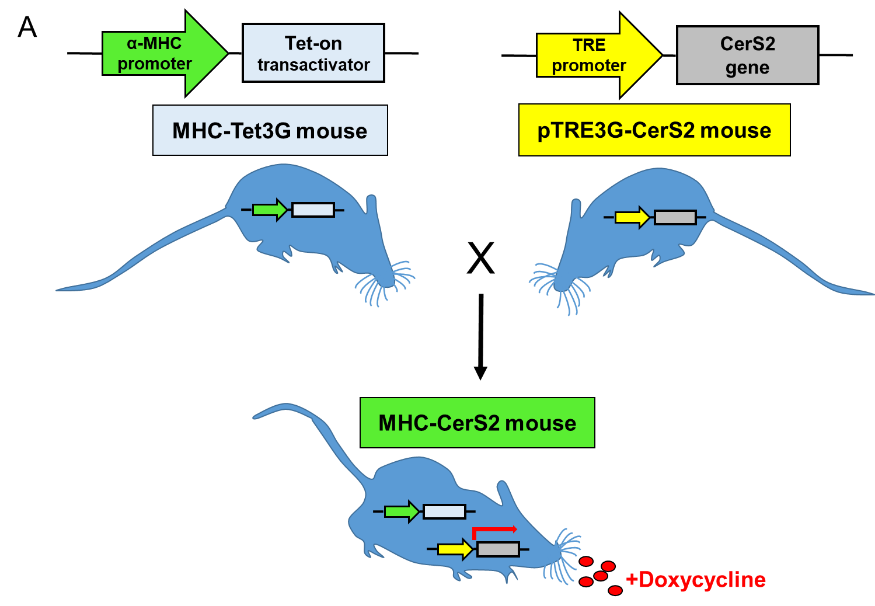


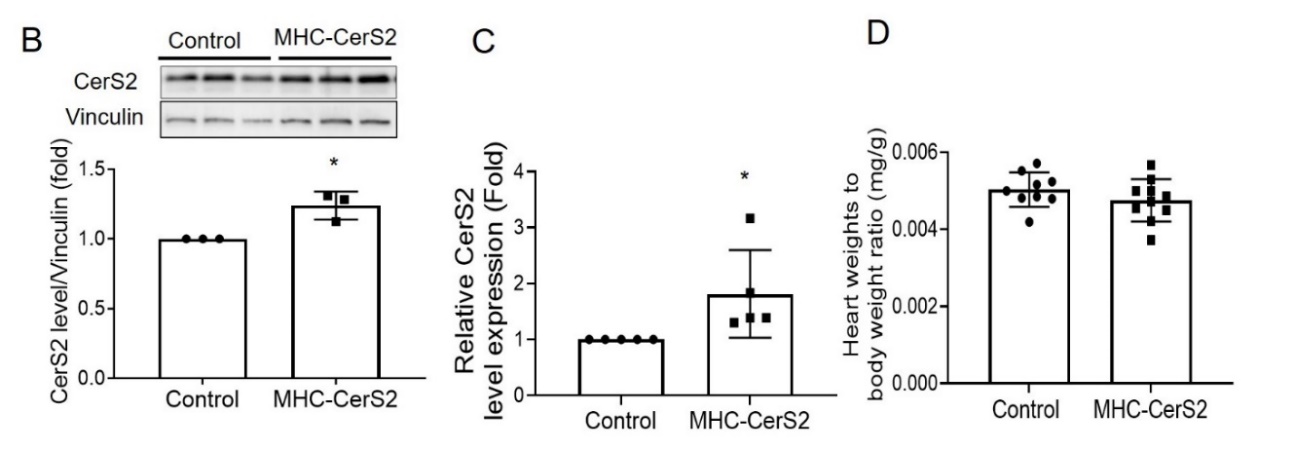


**Fig. S2: A)** Scheme for generating Tet on-controlled CerS2 overexpression mouse. The figure is adapted and modified from <https://www.takarabio.com/learning-centers/gene-function/inducible-systems/tet-inducible-systems/tet-systems-overview>. B,C) Increase CerS2 protein and gene expression in MHC-CerS2 mice after administered with doxycycline (0.545 mg/g) for one month was measured with western blot. Graph depicts mean value and standard deviation normalized to control. Significance was calculated with a t-test (p<0.05). D) Ratio of heart weight to body weight (mg/g) was measured after mice were euthanized by cervical dislocation and the hearts extracted.


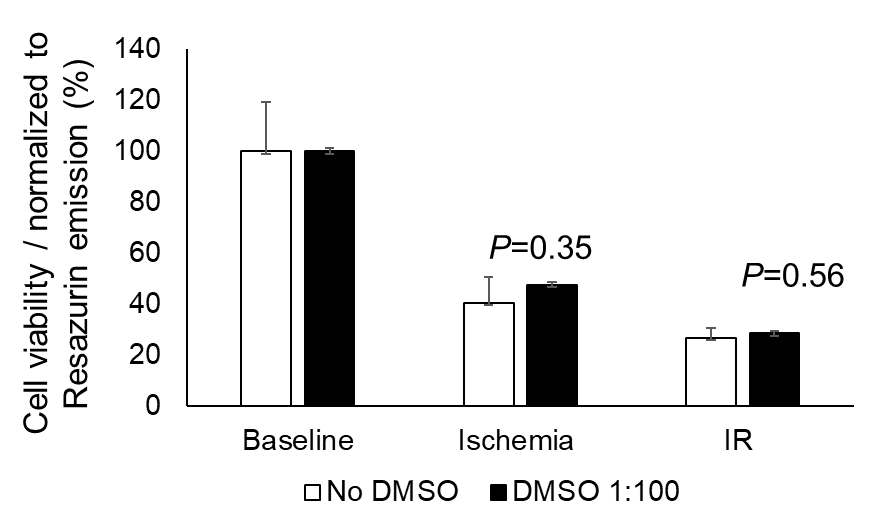


**Fig. S3.** Presto-Blue assay of viability of cardiomyocytes (CMs) incubated with and without DMSO during baseline, ischemia 6 hrs, and subsequent reperfusion for 16 hrs showed not a significant impact of the presence of DMSO in terms of viability compared to its absence. Comparisons between groups were made using paired t-test; *n=3*. Ischemia (I), reperfusion (R), and ischemia/reperfusion (IR).


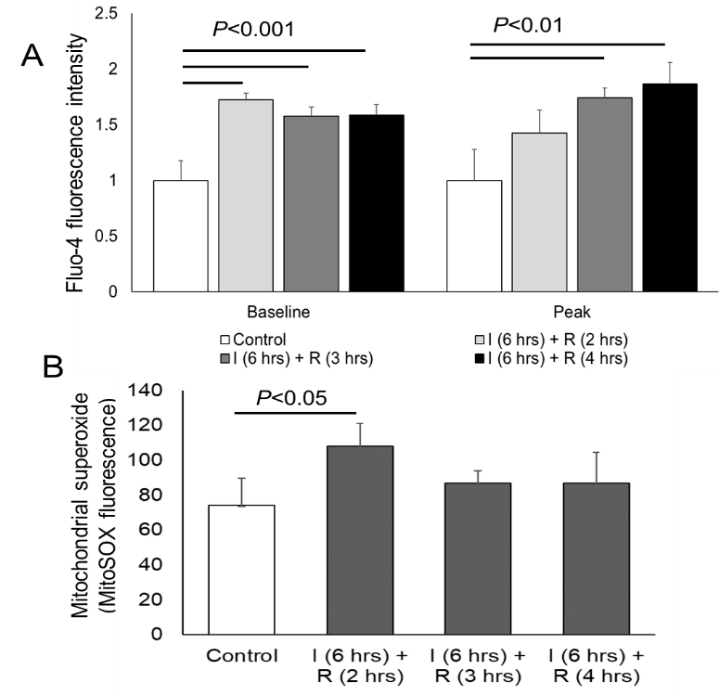


**Fig. S4.** Concentration of intracellular Ca^2+^ in resting (baseline) and contracting (peak) cardiomyocytes (CMs) and level of mitochondrial superoxide in control CMs and during ischemia/reperfusion with different reperfusion exposure times (2 hrs, 3 hrs, and 4 hrs). (A) Graphic representation of intracellular Ca^2+^ in resting (baseline) and contracting (peak) CMs detected with Fluo-4 dye in control and in samples undergoing ischemia 6 hrs followed by 2 hrs, 3 hrs, and 4 hrs of reperfusion. *n=3.* (B) Mitochondrial superoxide levels in control CMs and after CMs exposed to ischemia (6 hrs) followed by 2 hrs, 3 hrs and 4 hrs of *n=4*. Comparisons between groups were made using the unpaired t-test with relevant *p*-values are depicted reperfusion. Ischemia (I), reperfusion (R), and ischemia/reperfusion (IR).


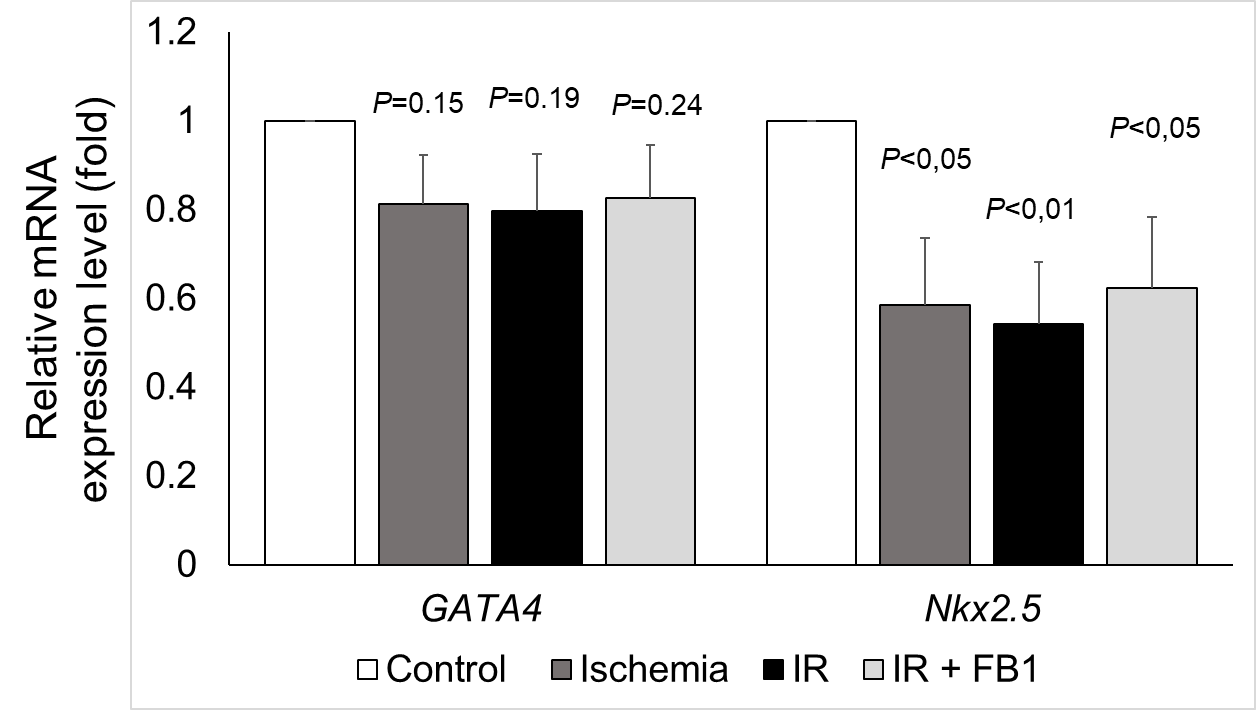


# Fig. S5. Gene expression levels of GATA4 and Nkx2.5 in CMs exposed to IR exposed to 6 hrs of ischemia and after 16 hrs of reperfusion with or without FB1 (50 µM).

# References

[1] K. Schonig, F. Schwenk, K. Rajewsky, H. Bujard, Stringent doxycycline dependent control of CRE recombinase in vivo, Nucleic Acids Res, 30 (2002) e134.
