# Supplementary material for: Inhibition of ceramide synthesis improves the outcome of ischemia/reperfusion injury in cardiomyocytes derived from human induced pluripotent stem cell
Source: Stem Cell Res Ther. 2025 Apr 18;16:190. doi: 10.1186/s13287-025-04340-3 (PMC12008854; doi:10.1186/s13287-025-04340-3)
Supplement: Supplementary file 2 — Supplementary Material 2 [file 13287_2025_4340_MOESM2_ESM.pptx]

## Slide 1
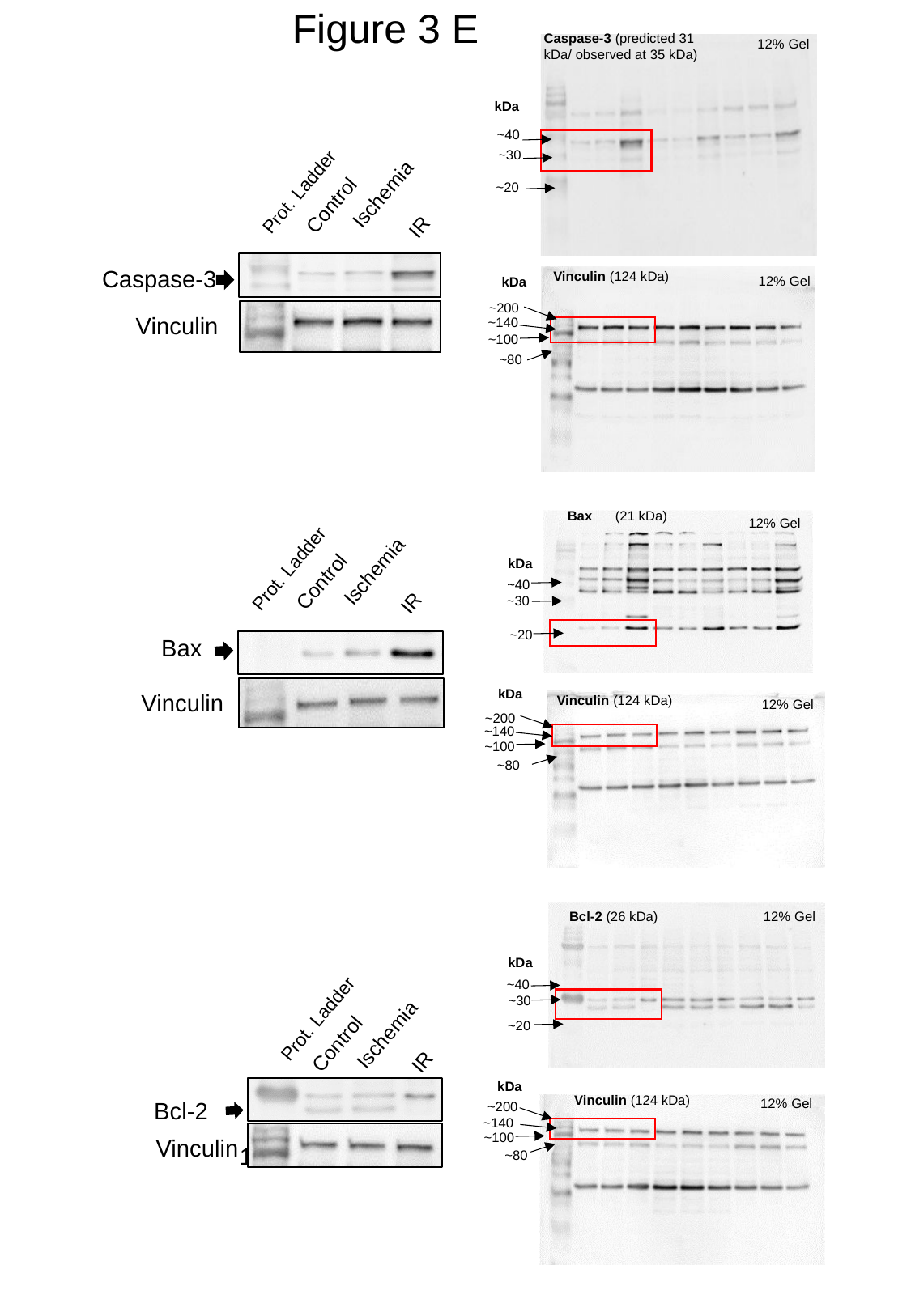

Figure 3 E
Caspase-3 (predicted 31 kDa/ observed at 35 kDa)
12% Gel
kDa
~40
~30
~20
Prot. Ladder
Ischemia
Control
IR
Caspase-3
Vinculin
Vinculin (124 kDa)
12% Gel
kDa
~200
~140
~100
~80
Prot. Ladder
Ischemia
Control
IR
Bax
Vinculin
Bax
(21 kDa)
12% Gel
kDa
~40
~30
~20
kDa
Vinculin (124 kDa)
12% Gel
~200
~140
~100
~80
Bcl-2 (26 kDa)
12% Gel
kDa
~40
~30
~20
Ischemia
Control
IR
Bcl-2
Vinculin
12 % Gel
Prot. Ladder
kDa
Vinculin (124 kDa)
12% Gel
~200
~140
~100
~80

## Slide 2
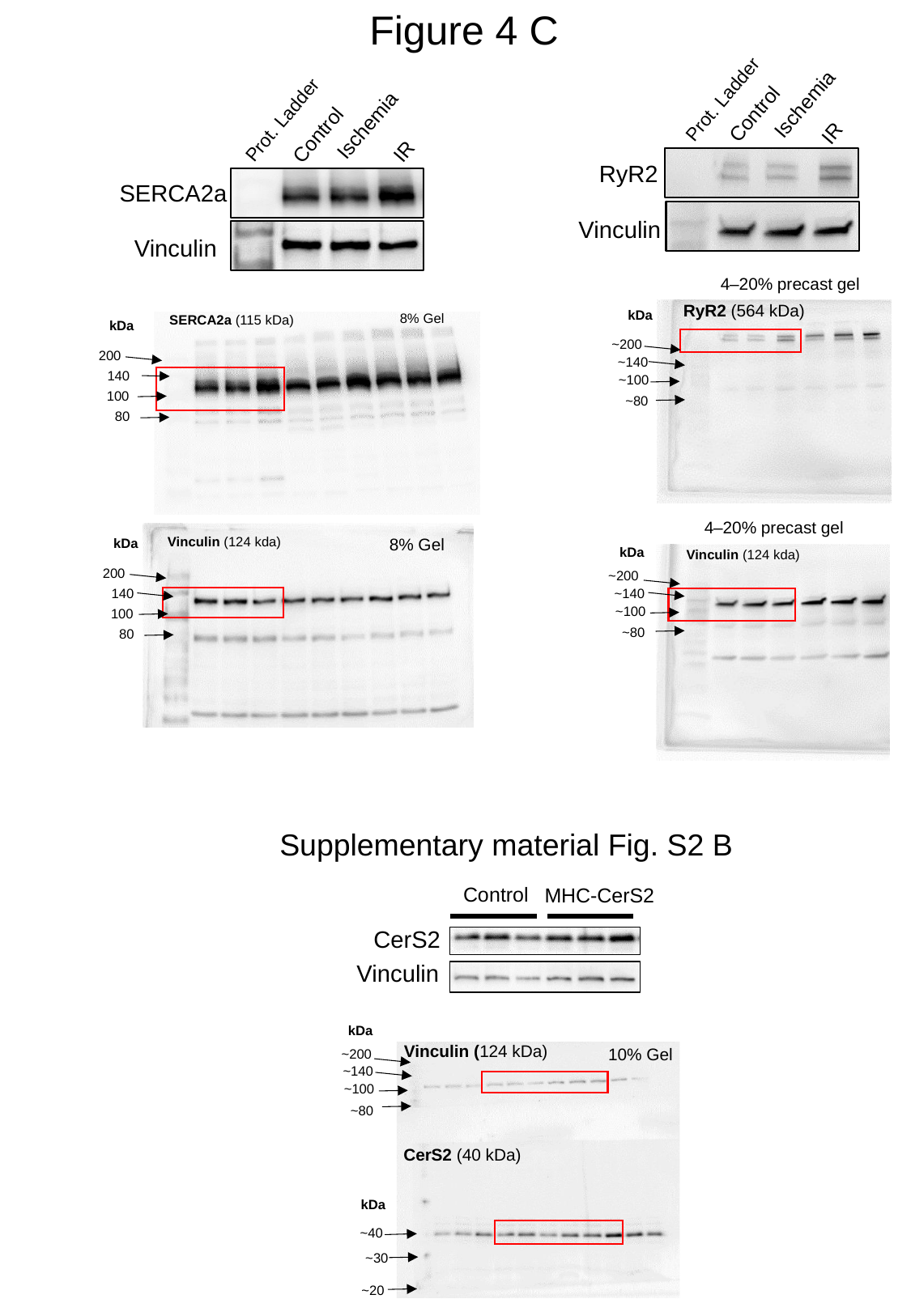

Figure 4 C
Prot. Ladder
Ischemia
Control
IR
RyR2
Vinculin
Prot. Ladder
Ischemia
Control
IR
SERCA2a
Vinculin
4–20% precast gel
RyR2 (564 kDa)
kDa
~140
~100
~80
8% Gel
SERCA2a (115 kDa)
kDa
200
140
100
80
~200
4–20% precast gel
kDa
Vinculin (124 kda)
~200
~140
~100
~80
Vinculin (124 kda)
8% Gel
kDa
200
140
100
80
Supplementary material Fig. S2 B
Control
MHC-CerS2
CerS2
Vinculin
kDa
Vinculin (124 kDa)
10% Gel
~200
~140
~100
~80
CerS2 (40 kDa)
kDa
~40
~30
~20

## Slide 3
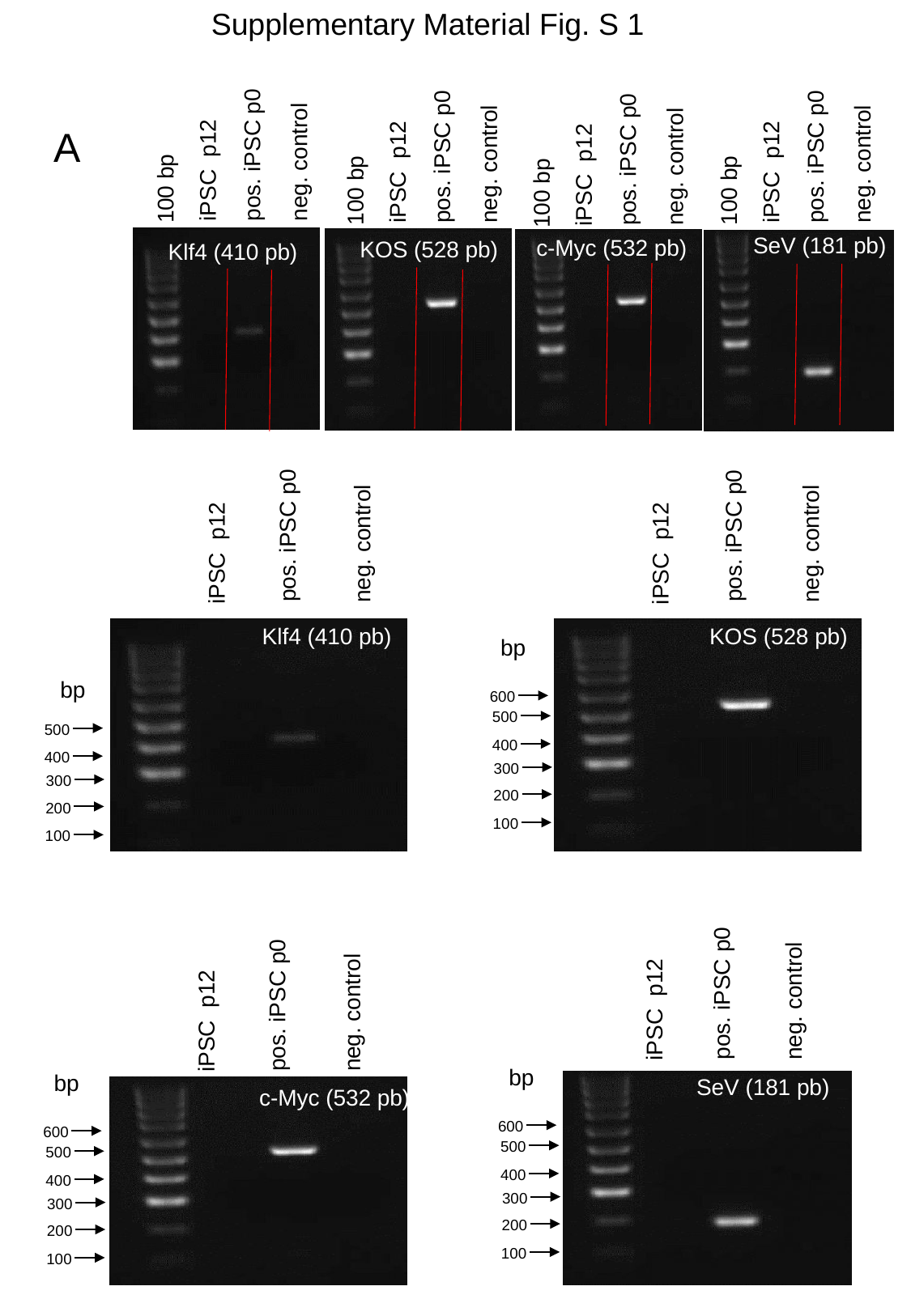

Supplementary Material Fig. S 1
A
pos. iPSC p0
pos. iPSC p0
pos. iPSC p0
pos. iPSC p0
neg. control
neg. control
neg. control
neg. control
iPSC p12
iPSC p12
iPSC p12
iPSC p12
100 bp
100 bp
100 bp
100 bp
SeV (181 pb)
c-Myc (532 pb)
KOS (528 pb)
Klf4 (410 pb)
pos. iPSC p0
neg. control
iPSC p12
KOS (528 pb)
bp
600
500
400
300
200
100
pos. iPSC p0
neg. control
iPSC p12
Klf4 (410 pb)
bp
500
400
300
200
100
pos. iPSC p0
neg. control
iPSC p12
SeV (181 pb)
bp
600
500
400
300
200
100
pos. iPSC p0
neg. control
iPSC p12
c-Myc (532 pb)
bp
600
500
400
300
200
100

## Slide 4
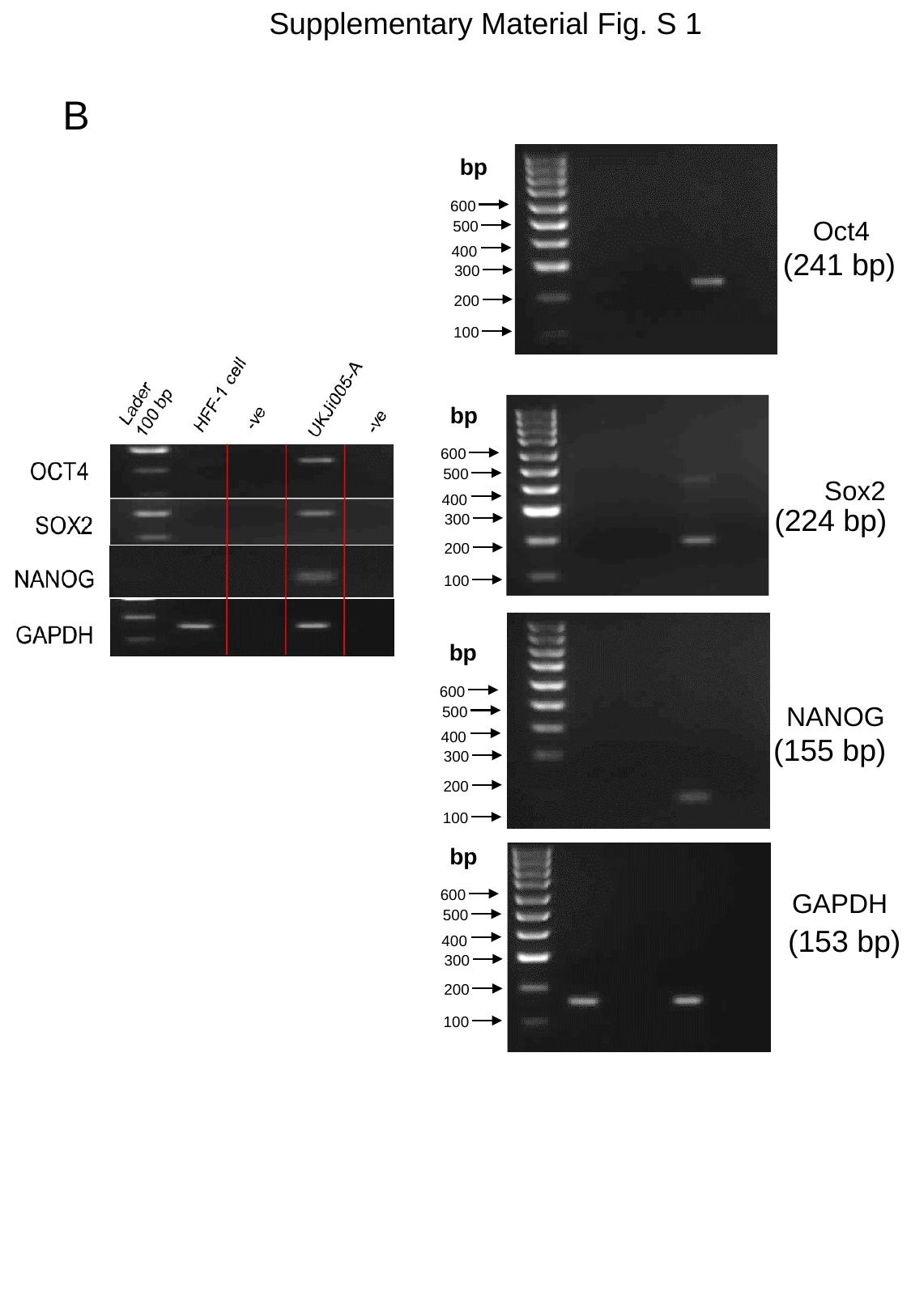

Supplementary Material Fig. S 1
B
bp
600
Oct4
500
400
(241 bp)
300
200
100
bp
600
500
Sox2
400
(224 bp)
300
200
100
bp
600
NANOG
500
400
(155 bp)
300
200
100
bp
600
GAPDH
500
(153 bp)
400
300
200
100
